# Anthropogenic Landscape Alteration, but Not Urbanization, Influences Non‐Adaptive Evolution in Common Milkweed ( *Asclepias syriaca* L.)

**DOI:** 10.1002/ece3.71250

**Published:** 2025-04-18

**Authors:** Sophie T. Breitbart, Marc T. J. Johnson, Helene H. Wagner

**Affiliations:** ^1^ Department of Ecology and Evolutionary Biology University of Toronto Toronto Ontario Canada; ^2^ Department of Biology University of Toronto Mississauga Mississauga Ontario Canada; ^3^ Centre for Urban Environments University of Toronto Mississauga Mississauga Ontario Canada

**Keywords:** effective population size, gene flow, genetic differentiation, genetic diversity, genetic drift, urbanization

## Abstract

Urbanization can alter mating and dispersal, with consequences for non‐adaptive evolution in populations. Potential outcomes vary widely due to the heterogeneity of urban landscapes and the diverse life history strategies of taxa. Furthermore, it is unclear how plants, which are significantly understudied in this context, are impacted. To better understand how urbanization influences non‐adaptive evolution in a native plant of conservation importance, we analyzed patterns of neutral genetic variation in common milkweed ( *Asclepias syriaca* ). From 256 individuals sampled across 122 locations throughout the Greater Toronto Area, Canada, we created two datasets of 2,835 and 972 single nucleotide polymorphisms through genotype‐by‐sequencing. Genetic diversity and effective population size *N*_*e*_ were mostly consistent between urban and rural habitats. Genetic differentiation between urban and rural habitats was low, and samples originated from a single genetic population. Demographic analysis indicated that *N*_*e*_ decreased by > 99% within the past 800 years, with the rate of loss accelerating over time. These findings suggest that this *A. syriaca* population was little affected by the transition from rural to urban habitat; rather, anthropogenic activity prior to urbanization, such as precontact Indigenous inhabitation and colonial settlement, had observable effects on population demography. This study demonstrates how anthropogenic factors can modify the degree to which urbanization impacts evolution and emphasizes the importance of contextualizing results with demographic, ecological, and cultural histories.

## Graphical abstract

We examined how urban environments influence neutral genetic variation in a native plant of conservation importance, common milkweed ( *Asclepias syriaca* ), in the Greater Toronto Area, Canada. We found little evidence that urbanization affected non‐adaptive evolutionary processes in this population. Instead, our results suggest that anthropogenic activity prior to urbanization, such as precontact Indigenous inhabitation and colonial settlement, had observable effects on population demography.

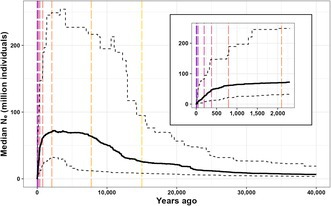

## 1 Introduction

The fast‐paced growth of urban areas is intensely altering ecosystems worldwide. Urban environments are typically characterized by factors including extensive habitat fragmentation, impervious surface coverage, increased temperature, and high human population density (Grimm et al. 2008; McDonnell and MacGregor‐Fors 2016). These factors can strongly shape how organisms inhabit and move through the urban landscape (Hamer and McDonnell 2008; Gallo et al. 2022; Youngsteadt and Keighron 2023). In turn, altered dispersal patterns can influence non‐adaptive evolutionary processes of gene flow and genetic drift within and between populations (Storfer et al. 2010; Miles et al. 2019). However, due to the diverse life histories of urban‐dwelling species and the heterogeneous nature of urban landscapes, it is difficult to predict the effects of urbanization on the genetic differentiation and diversity of natural populations.

**FIGURE 1:**
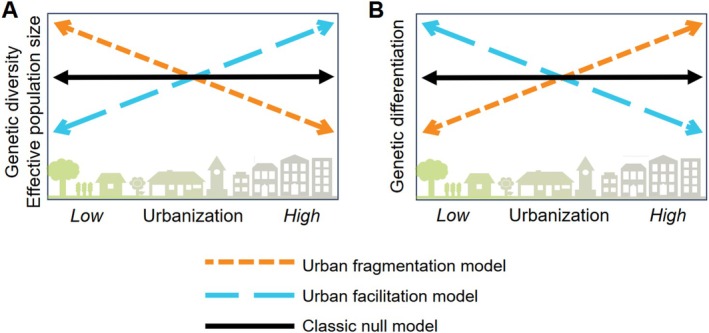
Predictions for how the urban fragmentation model, urban facilitation model, and classic null model influence (A) genetic diversity and effective population size, and (B) genetic differentiation within populations.

There are three conceptual models that predict how urban environments, compared to nonurban environments, affect non‐adaptive evolutionary processes in populations: the urban fragmentation model, the urban facilitation model, and a null model (Miles et al. 2018, 2019) (Figure 1). The urban fragmentation model predicts decreased dispersal throughout the urban landscape, which lowers gene flow between populations and results in increased habitat fragmentation and population isolation. In turn, these processes intensify the random loss and fixation of alleles caused by genetic drift (Barrett and Charlesworth 1991; Saccheri et al. 1996; Fowler and Whitlock 1999). Thus, the urban fragmentation model predicts lower genetic diversity within, and increased genetic differentiation among, urban populations (Johnson and Munshi‐South 2017; Miles et al. 2019). The urban facilitation model predicts the opposite effect. Under this model, increased dispersal in urban environments would weaken genetic drift and increase gene flow, as observed in a tropical tree (Noreen and Webb 2013) and bats (Richardson et al. 2021). Lastly, urban areas may not impact dispersal differently than nonurban environments, yielding negligible differences in genetic diversity and spatial genetic structure between urban and nonurban areas. This situation exemplifies a null model (henceforth, the “classic null model”), which has been observed in relatively mobile species including birds (Schmidt et al. 2020) and bumblebees (Theodorou et al. 2018). A specific type of null model is characterized by anthropogenic disturbance that predates urbanization, rather than dispersal. Under this “pre‐urbanization null model”, non‐adaptive evolutionary processes are expected to change before modern urbanization in response to longer‐term human settlement, shaping genomic variation in response. For instance, it has been hypothesized that demographic changes in two frog species were largely driven by events that occurred prior to modern urbanization, including agriculture (Wei et al. 2021) and European colonialism (Moran et al. 2024). While specific traits such as dispersal ability may inform how urbanization influences non‐adaptive evolution, it remains especially challenging to predict impacts on species that disperse with the help of animals or abiotic vectors (e.g., wind), which is common in plants.

Previous research on our study organism, common milkweed ( *Asclepias syriaca* ), suggests that non‐adaptive evolutionary processes may shape its genotypic and phenotypic patterns in the Greater Toronto Area (GTA). For example, an observational study showed that urbanization influenced phenotypic divergence in multiple reproductive traits in *A. syriaca* , as well as the community structure of its pollinators (Breitbart et al. 2023b). However, urbanization did not influence genetic divergence in > 20 phenotypic traits assessed in a common garden experiment of seeds from the same urbanization gradient (Breitbart et al. 2023a). These results indicate that the previously observed phenotypic divergence was consistent with phenotypic plasticity, as opposed to genetic divergence, and that adaptation by natural selection across the GTA was unlikely to have occurred in those traits at that time. Additionally, over larger spatial and temporal scales, demographic changes have been detected in *A. syriaca* . Throughout its native and core ranges, *A. syriaca* experienced demographic expansions concurrent with the last glacial maximum and pre‐industrial agriculture (1751–1899 ad), but not bottlenecks concurrent with industrial agriculture (1945–2015 ad) (Boyle et al. 2023). Large‐scale studies have also shown low genetic differentiation among local populations of *Asclepias* spp. (Sussman 2017; Boyle et al. 2023) and inferred a single, nearly panmictic population of *A. syriaca* across the species' North American range (Boyle et al. 2023). Thus, the apparent lack of genetic divergence of *A. syriaca* in the GTA may have primarily resulted from non‐adaptive evolutionary processes—specifically, high gene flow between patches and/or weak genetic drift within patches. Moreover, assessing how urban areas like the GTA impact neutral genetic variation is critical for understanding how urbanization influences the evolutionary potential of populations.

Here, we examined how urbanization affects non‐adaptive evolution in *A. syriaca* , a native plant of conservation importance. We genotyped plants sampled across urban and rural habitats within the GTA using genotyping‐by‐sequencing. To determine whether our data best supported the urban facilitation model, the urban fragmentation model, or a null model, we asked two questions: (1) How does urbanization influence genetic diversity and population genetic structure? (2) How does historical anthropogenic change impact effective population size (*N*_*e*_) and historical changes in *N*_*e*_? Answering these questions will provide insight into how urbanization influences genetic drift and gene flow in this native plant of conservation concern.

## 2 Methods

### 2.1 Study System

Common milkweed, *Asclepias syriaca* L., is an herbaceous perennial native to eastern North America. Common milkweed typically grows in open areas like old fields, roadsides, and forest edges in discrete patches of up to thousands of clonal ramets branching from rhizomes that can persist for decades (Bhowmik and Bandeen 1976; Wilbur 1976). Public participation in conservation‐oriented campaigns has increased plant density in urban and suburban environments, where populations tend to be smaller and inhabit parks, railway corridors, roadsides, lawns, and gardens (Shahani et al. 2015; Johnston et al. 2019; Breitbart et al. 2023b). Plants are mostly outcrossed, with pollination facilitated by over 20 generalist and specialist insects, including *Apis mellifera* , *Bombus* spp., and *Halictidae* spp. (MacIvor et al. 2017; Baker and Potter 2018; Breitbart et al. 2023b). The ovules within each fruit (follicle) are pollinated by a single pollen sac (pollinium), resulting in hundreds of wind‐dispersed, full‐sibling seeds. Reproduction can also occur asexually through underground rhizomes that spread vegetatively and produce clonal ramets (Bhowmik and Bandeen 1976). Common milkweed is of high conservation importance because it is the main host of the iconic yet endangered migratory monarch butterfly ( *Danaus plexippus* ), which requires milkweeds (*Asclepias* spp.) for reproduction and survival (U.S. Fish and Wildlife Service 2020).

Common milkweed is a useful system for studying the effects of urbanization on non‐adaptive evolutionary processes. For instance, urbanization can alter the species' diverse pollinator communities (Breitbart et al. 2023b), and pollen dispersal behavior varies among its pollinators (Kephart 1983; Howard and Barrows 2014; Gustafson et al. 2023). As a largely self‐incompatible obligate outcrosser (Wyatt and Broyles 1994), variable pollination efficiency could impact pollen flow within and among populations. How and where the species' wind‐dispersed seeds travel throughout diverse urban landscapes may also be influenced by multiple factors associated with urban environments, such as altered wind patterns, turbulence, and local adaptation (Cheptou et al. 2008; Von Der Lippe and Kowarik 2008; Kowarik and von der Lippe 2011). Thus, altered pollen and seed flow could yield consequences for gene flow and genetic drift in common milkweed.

### 2.2 Field Sampling

**FIGURE 2:**
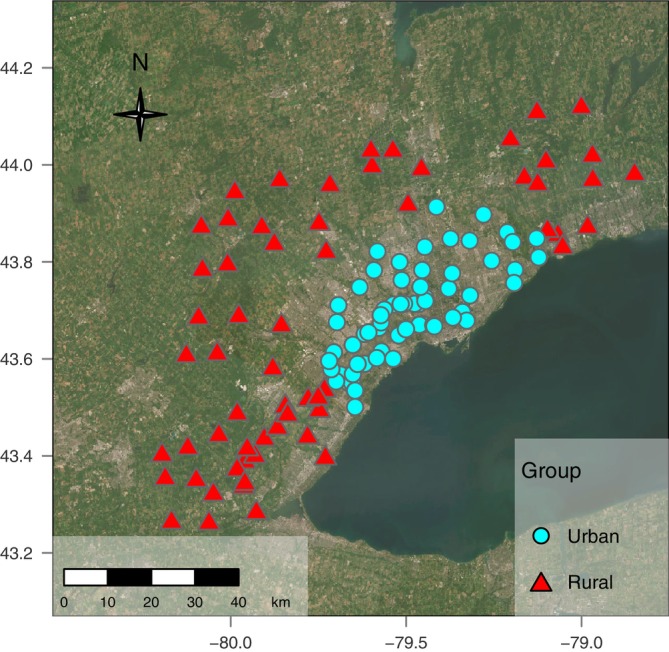
Map of 124 common milkweed sampling sites throughout the Greater Toronto Area (GTA). Red triangles (*n* = 61) and blue circles (*n* = 63) indicate classification as “rural” and “urban”, respectively, based on distance from the city center (as opposed to urbanization score, shown in Figure S3). The ESRI world imagery basemap shows urban and suburban areas in light gray, nonurban agricultural and forested areas in green, and Lake Ontario in blue (ESRI world imagery basemap 2023). Tiles Esri—*Source:* Esri, i‐cubed, USDA, USGS, AEX, GeoEye, Getmapping, Aerogrid, IGN, IGP, UPR‐EGP, and the GIS user community.

We collected leaves from 124 sampling sites distributed throughout the GTA's urbanization gradient during the summer of 2018 (Figure 2, Figure S1). We used two sampling designs in an effort to capture broad‐scale and local genetic variation. For the first design, we sampled one leaf from each of 74 urban or rural sampling sites spaced in cells of 2.5 km × 2.5 km (urban) or 10 km × 10 km (rural). The different sample densities were chosen to obtain approximately equal representation of urban and rural sampling sites. To capture smaller‐scale genetic variation along the urbanization gradient, we sampled 50 additional sites following a transect (Breitbart et al. 2023a). We sampled one leaf from each of 1–5 ramets per sampling site, with ramets separated by > 3 m to prevent resampling the same genetic individual, with variable sampling due to ramet availability. Overall, we sampled one individual from 79 sampling sites and between 2 and 5 individuals from the remaining 45 sites (Figure S1). Sampling sites within grid cells or along the transect were chosen haphazardly in diverse environments including parks, farmland buffers, railway corridors, roadsides, and residential gardens. Leaves were placed on ice in a cooler and transferred to a −20°C freezer until DNA extraction.

### 2.3 Urbanization Metrics

We quantified urbanization using two proxies as discussed in Breitbart et al. (2023b): Distance from the City Center and Urbanization Score. To calculate the first metric of urbanization, we measured the distance from each sampling site to the Toronto urban center (43.6563, −79.3809) with the R package *geosphere* v1.5.18 (Hijmans 2021). Distance to Toronto's urban center correlates with factors characteristic of urban environments including canopy and impervious surface coverage and has been found to be associated with ecological and evolutionary change in other plant species (Johnson et al. 2018; Rivkin et al. 2020; Murray‐Stoker and Johnson 2021). For the second metric, we used the UrbanizationScore software (Czúni et al. 2012; Seress et al. 2014; Lipovits et al. 2015) to calculate a metric capturing the composition of vegetation, buildings, and roads in a 1 km radius around each sampling site. Urbanization scores ranged from −3.56 (least urban) to 3.88 (most urban) and were highly correlated with sampling sites' distances to the city center (ANOVA: *F*_1,160_ = 264.642, *p* < 0.001, *R* ^2^_adj_ = 0.621) (Figure S2). Each site was classified twice as either urban or rural, with each classification based on one metric of urbanization. Based on Distance from the City Center, a sampling site was classified as “urban” if the distance from the city center was ≤ 30 km. Based on the Urbanization Score, a site was classified as “urban” if its score was > 0 (Figure 2, Figure S3).

### 2.4 DNA Extraction and Sequencing

We extracted DNA from 263 leaf samples. This involved grinding 15–20 mg freeze‐dried leaf tissue (excluding the midrib) for 2 × 3 min at 2000 rpm in a Qiagen TissueLyser and extracting DNA using the Qiagen DNeasy Plant Kit with slightly modified instructions to increase yield (Supporting Information). We evaluated DNA quantities with a Qubit Fluorometer dsDNA High Sensitivity Assay and ran all samples on a 1.5% agarose gel to assess quality. Samples were dried in 96‐well half‐skirted plates at 45°C for 1.5 h in an Eppendorf Vacufuge Concentrator, then shipped to the Elshire Group (Palmerston North, New Zealand) for library preparation and sequencing using genotyping‐by‐sequencing (Elshire et al. 2011). The data were generated following a modified version of the protocol described in Elshire et al. (2011). Briefly, 100 ng of genomic DNA was plated with “barcode” and “common” adapters, then digested for 2 h at 75°C with the restriction enzyme PstI. Adapters were ligated by adding 30 mL of a solution containing 1.66x ligase buffer with ATP and T4 ligase to each sample well, then incubating at 22°C for 1 h and heating to 65°C for 30 min to inactivate the T4 ligase. Samples were combined, purified using the QIAquick PCR Purification Kit, and eluted to a final volume of 50 μL. Restriction fragments were then amplified for 18 PCR cycles after adding the primers 5′AATGATACGGCGACCACCGAGATCTACACTCTTTCCCTACACGACGCTCTTCCGATCT and 5′CAAGCAGAAGACGGCATACGAGATCGGTCTCGGCATTCCTGCTGAACCGCTCTTCCGATCT. The resulting libraries were purified again as described above, then evaluated to ensure that the majority of DNA fragments were between 170–350 bp. Finally, an Illumina HiSeq XTen was used to generate reads from all 263 samples, yielding a total of 1.109 billion 150 bp paired‐end sequences.

### 2.5 Calling Single Nucleotide Polymorphisms (SNPs)

We demultiplexed the raw sequences with Ax v0.3.3 (Murray and Borevitz 2018) using a maximum hamming distance mismatch of zero, then trimmed adaptors and discarded reads with uncalled bases using Trim Galore v0.6.7 (Krueger et al. 2023). We analyzed read quality for each sample with FastQC (Andrews 2010), then generated summary reports with MultiQC (Ewels et al. 2016) and removed two samples due to low quality. Consequently, one sampling site was eliminated. We used fastp v0.23.1 (Chen et al. 2018) to trim reads to a uniform length of 110 bp and remove reads with Phred quality scores < 20 (i.e., base call accuracy < 99%). We used BWA v0.7.17 (Li and Durbin 2009) to index the *A. syriaca* reference genome (Straub et al. 2011), a step required to increase the computational efficiency of the subsequent alignment process, then aligned and assembled reads to the genome. We converted SAM files to BAM format with SAMtools v.1.16.1 (Li et al. 2009), evaluated sequence quality with BamTools v2.5.1 (Barnett et al. 2011), and sorted the files with SAMtools. We identified five pairs of individuals with 25%–75% identity (i.e., at least half‐siblings) with PLINK v1.9 (Chang et al. 2015), then removed one individual from each of the five pairs from the dataset, which eliminated one sampling site (Table S1). Next, we created a genome‐wide SNP library with Stacks v2.62 (Catchen et al. 2013) for the remaining 256 individuals from 122 sampling sites. Unless otherwise indicated, we used the default options for the aforementioned steps. In total, we retained 48,349 loci with an average depth of 403.3× (SD = 169.3×) and an average of 68.7 nucleotide sites per locus.

We used the Stacks *populations* script to filter the library with two sets of criteria to create datasets for analyses with different purposes and generate summary statistics for each dataset. First, we created a SNP dataset (#1) for analyses that are robust to rare variants and missing data (pertaining to the genetic diversity analyses in Question 1 except for the *F*_*IS*_ analyses, and Question 2). These criteria maintained minor alleles with low frequencies (rare variants) by setting the minimum minor allele frequency to 2/256 (bash flag *‐‐mmaf* = 0.0078125) to ensure that a locus would be analyzed if it were present in at least two individuals and retained loci with valid data for at least 50% of individuals (*‐‐R* = 0.5). For this dataset, we retained an average of 73.79 nucleotide sites (variant and invariant) per locus, with 2,835 variant sites within 4,781 loci. Secondly, we used strict filtering criteria to create another SNP dataset (#2) (pertaining to the population genetic structure analyses in Question 1, plus the *F*_*IS*_ analyses). These criteria removed minor alleles with low frequencies by setting the minimum minor allele frequency to 5% (*‐‐mmaf* = 0.05) and retained loci with valid data for at least 75% of individuals (*‐‐R* = 0.75). For this dataset, we retained an average of 73.74 nucleotide sites per locus, with 972 variant sites within 3519 loci. For both datasets, we retained only the first SNP per locus (*‐‐write‐single‐snp*) to avoid nonindependence among SNPs in the same locus due to linkage disequilibrium. Summary statistics for each dataset were calculated with Stacks and included the following for each nucleotide position and sampling site (i.e., averaged among 1–5 genotyped individuals): inbreeding coefficient (*F*_*IS*_) (Wright 1949), expected (*H*_*e*_) and observed heterozygosity (*H*_*o*_), and nucleotide diversity (*π*) (Nei and Li 1979) (Tables S2–S5).

### 2.6 Question 1: How Does Urbanization Influence Genetic Diversity and Population Genetic Structure?

#### 2.6.1 Genetic Diversity

First, we determined the effects of a gradient of urbanization on nucleotide diversity and inbreeding. We treated sampling‐site level *π* and *F*_*IS*_ as response variables in the general linear mixed models shown in Equation (1), which were fitted with the R package *glmmTMB* v1.1.7 (Brooks et al. 2017) using maximum likelihood:

(1) Response variable~Distance from the City Center OR Urbanization Score+Individuals+Error

In these models, *Distance from the City Center* and *Urbanization Score* are continuous fixed effects. *Individuals* is a fixed effect accounting for the number of genotyped individuals per sampling site. We excluded 77 sampling sites with a single genotyped individual from this analysis. We used the “Anova” function from the R package *car* v3.1.2 (Fox and Weisberg 2019) to perform ANOVA with type II sums‐of‐squares (Langsrud 2003). We inspected model diagnostics with the R package *performance* v0.10.4 (Lüdecke et al. 2021) to confirm assumptions of normality, homoscedasticity, independence, and linearity. All R analyses were performed in R v4.3.1 (R Core Team 2020).

To compare levels of genetic diversity within and between groups (urban vs. rural), we ran Stacks *populations* program scripts with the aforementioned parameters to obtain mean *π* and *F*_*IS*_ for each group (urban vs. rural), as well as for the entire population (i.e., for all sampling sites combined). Compared to the first *populations* scripts, this step involved creating three groups—urban, rural, and the entire population—and removing sampling site substructure so that all individuals within the groups were treated as part of a single population. We then used the R package *pegas* v1.2 (Paradis 2010) to calculate Watterson's θ, another estimate of nucleotide diversity (Watterson 1975), for each urban and rural habitat and the entire population. Examining two estimates of nucleotide diversity allowed us to more thoroughly understand the genetic diversity: Watterson's θ is influenced by the number of segregating sites while *π* conveys the expected heterozygosity of those segregating sites.

Next, we calculated Tajima's D (Tajima 1989) in 100 kb windows across the genome with the program vcftools v0.1.16 (Danecek et al. 2011) for the urban and rural groups, and the entire population. Again, we removed sampling site substructure so that all individuals within the groups were treated as part of a single population. This test allowed us to test the null hypothesis that each group is evolving neutrally, against the alternative hypothesis that it is experiencing demographic change (Kimura 1983). A value of Tajima's *D* that is significantly different from zero could indicate a sizable population expansion (*D* < 0) or contraction (*D* > 0) (Tajima 1989). We then used the R package *stats* (R Core Team 2020) to perform a single‐sample *t*‐test for each group to assess whether Tajima's *D* was significantly different from zero. In addition, we performed a two‐sample *t*‐test to test for a significant difference in mean *D* between urban and rural groups.

#### 2.6.2 Population Genetic Structure

We used three approaches to determine the number of genetic clusters: Principal Component Analysis (PCA), Discriminant Analysis of Principal Components (DAPC) (Jombart et al. 2010), and the inference of genetic structure in a geographically distributed population using the R package *conStruct* v1.0.5 (Bradburd et al. 2018) (Supporting Information). We first used PCA and DAPC since these tools work quickly and do not require the quantification of hypothesized clusters a priori, making them effective for exploratory analysis. Furthermore, we used DAPC because it maximizes among‐group variation and minimizes within‐group variation; in contrast, PCA is not biased by this feature. The spatially explicit conStruct analysis complemented the other clustering methods but differed by allowing for isolation‐by‐distance (i.e., modeling how allele frequency covariances decay spatially) and relaxing the prior methods' assumptions that samples were spatially independent. This analysis was used to distinguish whether *A. syriaca* exhibited discrete clusters or continuous genetic variation in allele frequencies. As the PCA and DAPC results both suggested a single genetic population, conStruct models were run testing a small range of clusters (*K* = 1–5). We tested for both continuous and discrete clusters by fitting spatial and nonspatial models in conStruct, each with 10,000 iterations and 5 Markov‐chain Monte Carlo chains. We performed cross‐validation for each model using 8 repetitions per K, 10,000 iterations per repetition, and a training proportion of 0.7. We identified whether the best model was spatial or nonspatial by evaluating which model showed the highest predictive accuracy for each value of K. To identify the optimal K, we assessed the predictive accuracy of each layer (i.e., hypothesized genetic population) and contribution within the optimal model to the total model covariance.

Next, we assessed how urbanization influenced genetic differentiation among sampling sites within groups, and between the urban and rural groups. We calculated Hudson's F_ST_ for each pair of sampling sites following the equation provided in Bhatia et al. (2013), then calculated the average pairwise F_ST_ between sampling sites within and between groups (i.e., urban–urban, rural–rural, and urban–rural pairs). Sampling sites with a single genotyped individual were excluded since calculating Hudson's F_ST_ would require dividing by zero. We then used an Analysis of Molecular Variance (AMOVA) framework to complement this approach. This framework allowed us to further investigate how genetic variation was partitioned among hierarchical groups without making assumptions about Hardy–Weinberg equilibrium (Excoffier et al. 1992). We used the “poppr.amova” function from the R package *poppr* v2.9.4 (Kamvar et al. 2014) to perform the AMOVA using Euclidean distances of allele frequencies, then used the function “randtest” from the package *ade4* v1.7.22 (Dray and Dufour 2007) to test the significance of each variance component. We also performed AMOVA by excluding sampling sites with a single genotyped individual and found that the results were qualitatively identical (Table S6).

We used Permutational Analysis of Variance (PERMANOVA) (Anderson 2001) and Permutational Multivariate Analysis of Dispersion (PERMDISP) implemented in the R package *vegan* v2.6.4 (Oksanen et al. 2019) to explore whether the means of the pairwise genetic distances (PERMANOVA) or their variances (PERMDISP) differed between urban and rural groups (Supporting Information). We used the functions “adonis2” and “betadisper” for the PERMANOVA and PERMDISP analyses, respectively. Pairwise genetic distances were calculated as Euclidean distances of allele frequencies.

Lastly, we used two methods to compare spatial genetic structure between the urban and rural groups. For each method and group, we performed a main analysis that restricted each sampling site to include a single genotyped individual to control for variable sampling depth within our study area, and an additional analysis that included all genotyped individuals (Supporting Information). We tested for positive spatial autocorrelation consistent with isolation‐by‐distance by creating Mantel correlograms with the “mantel.correlog” function from the R package *vegan*, then used the R package *memgene* v1.0.2 (Galpern et al. 2014) to test for the presence of cryptic spatial genetic variation within the urban and rural habitats (Supporting Information). In contrast to Mantel correlograms, the latter method employs a multivariate regression approach with Moran eigenvector maps as orthogonal predictors to identify the spatial component of genetic variation at multiple spatial scales. Hence, it can detect more complex spatial genetic structure than (spatially uniform) isolation‐by‐distance, such as isolation‐by‐resistance or the presence of barriers.

### 2.7 Question 2: How Does Historical Anthropogenic Change Impact Effective Population Size (*N_e_*) and Historical Changes in *N*_*e*_?

We inferred demographic history, separately for the urban and the rural groups and for the entire population, by modeling past changes of effective population size over time. First, we used the R package *vcf2sfs* v2.0 (Liu et al. 2018) to create a site frequency spectrum (SFS) for each group. Next, we used the SFS to reconstruct each group's demographic history using Stairway Plot v2 (Liu and Fu 2020). This tool was well‐suited for our study as it is applicable for genotype‐by‐sequencing datasets, does not require specifying a predefined demographic model (i.e., it is model‐flexible), and performs well when reconstructing recent demographic histories (Liu and Fu 2020). We assumed a mutation rate of 1.8 × 10^−8^ per site per generation based on a published rate from *Trifolium repens* (white clover) (Griffiths et al. 2019) and a two‐year generation time, using 67% of segregating sites used for training and 33% for testing with 200 bootstrap replicates. To assess the sensitivity of results to these assumptions, we repeated the analysis with a one‐year generation time and with mutation rates varying from 1.0 × 10^−8^—2.6 × 10^−8^ per site per generation (Supporting Information). We acknowledge that the algorithm underlying this analysis has additional assumptions that cannot be met using our dataset due to the life history traits of *A. syriaca* , including overlapping generations and uneven reproductive output associated with clonality (Montano 2016); thus, our results should be interpreted with this caveat.

To identify breakpoints between periods of constant rates of change in *c*, we assumed an exponential growth (or decay) model Equation (2) that describes the change in *N*_*e*_ between an initial point in time (*N*_*e*0_) and time *t* (*N*_*et*_) with rate *r* (growth: *r* > 0, decay: *r* < 0):

(2) $$N_{\mathrm{et}}=N_{e0}∙{\left(1+r\right)}^{t}$$

We linearized the relationship by taking the natural logarithm on both sides:

(3) $$\ln\left(N_{\mathrm{et}}\right)=\ln\left(N_{e0}\right)+t∙\ln\left(1+r\right)$$

Equation (3) implies that, when plotting ln(*N*_*et*_) against time *t*, a period with a constant rate of change will be characterized by a constant slope of *b* = ln(1 + *r*), whereas different periods would differ in their slope *b* due to a different rate of change, *r*. We used piecewise regression analysis (Muggeo 2003) with the R package *segmented* v2.1 (Muggeo 2008) to identify breakpoints between linear segments in the relationship between the median estimate (from the 200 bootstrap replicates) of ln(*N*_*et*_) and time *t* (in years before the field sampling year, 2018). We subsampled years systematically (every year 1–100, every 19th year between years 100–2,000, and every 130th year between years 2,000–15,000) for a total of 300 subsampled years. The function “segmented” requires the user to provide an initial list of breakpoints (as a starting point for the algorithm), which we identified iteratively by fitting an initial model for each of the three subsampling periods. The final piecewise regression model with 8 breakpoints was fitted to all 300 subsampled years (1–15,000). The function “segmented” returns standard errors for the breakpoint estimates (years) and, for each linear regression segment between two consecutive breakpoints, the estimate and the upper and lower limits of a 95% confidence interval for the slope coefficient *b*. We used Equation (4) to derive the corresponding estimate and 95% confidence interval for the growth rate *r* for each segment. Note that the sign is reverted to account for the backward modeling in time *t*:

(4) $$r=-1∙\left(e^{b}-1\right)$$

## 3 Results

### 3.1 Question 1: How Does Urbanization Influence Genetic Diversity and Population Genetic Structure?

#### 3.1.1 Genetic Diversity

Genetic diversity was mostly, but not completely, unaffected by urbanization. Nucleotide diversity *π* increased with increasing Distance from the City Center (*χ* ^2^ = 4.489, *p* = 0.034, *R* ^2^_m_ = 0.404) such that it was 9.2% lower in the most urban sampling sites, whereas *π* did not show a statistically significant change with Urbanization Score (*χ* ^2^ = 1.850, *p* = 0.174, *R* ^2^_m_ = 0.369) (Figure S4). Likewise, urbanization was not significantly associated with changes in *F*_*IS*_ using either predictor (Distance: *χ* ^2^ = 0.654, *p* = 0.419, *R* ^2^_m_ = 0.289; Urbanization Score: *χ* ^2^ = 0.047, *p* = 0.829, *R* ^2^_m_ = 0.279) (Figure S5).

Neither measure of nucleotide diversity (*π*, Watterson's θ), nor mean *F*_*IS*_, differed between urban and rural habitats (Table 1, Table S7). Tajima's D was negative and significantly different from zero for both groups (*p*‐values: Urban = < 0.001, Rural = < 0.001), but did not differ significantly between urban and rural habitats (Figure S6, Table S8).

**TABLE 1:** Genetic diversity, *F*_*IS*_, and Tajima's D calculated for the entire population and within urban and rural groups when urbanization was classified based on distance from the City Center. Genetic diversity is represented by Watterson's θ (θ_W_) and nucleotide diversity (*π*). Mean Tajima's D was calculated by averaging values from 100 kbp windows, and *p*‐values are shown for single‐sample *t*‐tests testing for deviations of Tajima's *D* from zero.

Habitat	θ_W_	*π*	*F*_*IS*_	Tajima's *D*	*p*
Entire Population	0.00131	0.00091	0.191	−0.275	< 0.001
Urban	0.00144	0.00091	0.194	−0.360	< 0.001
Rural	0.00153	0.00090	0.181	−0.363	< 0.001

#### 3.1.2 Population Genetic Structure

All methods used for identifying the number of genetic populations identified a single genetic population (Supporting Information, Figures S7–S9, Tables S9, S10). The spatial conStruct model with *K* = 1 best explained the population structure, suggesting that genetic variation changed continuously within a single genetic population.

Urbanization did not impact genetic differentiation between urban and rural habitats. Mean pairwise Hudson's F_ST_ was effectively zero for all subsets of sampling site pairs (urban–urban: −0.032; rural–rural: −0.017; urban–rural: −0.021), demonstrating low genetic differentiation among sampling sites regardless of habitat (Table S11). The AMOVA analysis supported these results, showing that < 1% of the total genetic variation existed among urban and rural groups (Tables S6 & S12). The most genetic variation was within sampling sites, both when all sampling sites were included (85.5%) and when only sampling sites with at least two individuals were included (99%).

The PERMANOVA and PERMDISP analyses suggested that sampling sites within urban and rural habitats exhibited comparable degrees of genetic differentiation. Distributions of pairwise genetic distances were not significantly different between the urban and rural habitats for either metric of urbanization (Table S13). While the variances of genetic distances were 5.5% higher in the urban habitat when urbanization was classified based on Distance from the City Center, this difference was only marginally significant and had a small effect size (*F*_1,254_ = 3.250, *p* = 0.072, *R* ^2^ = 0.013). There was no effect for Urbanization Score (*F*_1,254_ = 1.812, *p* = 0.180, *R* ^2^ = 0.007) (Table S14).

There was little spatial genetic structure at multiple spatial scales (Supporting Information). When urbanization was based on Distance from the City Center, the Mantel correlograms for the urban and rural groups did not detect positive spatial autocorrelation, suggesting a lack of isolation‐by‐distance (Figure S10, Table S15). The memgene analysis identified zero significant Moran eigenvector maps (MEM) in the urban habitat, signifying no detectable spatial structure in the genomic data for this group. While there were two significant MEM in the rural habitat, the variation explained by spatial patterns in this model was consistent with panmixia (*R* ^2^_adj_ = 0.01) (Figure S11, Table S16). Results for analyses that classified urbanization based on Urbanization Score and/or included all genotyped individuals are provided in the Supplement (Supporting Information, Figures S12–S18, Tables S17–S23).

### 3.2 Question 2: How Does Historical Anthropogenic Change Impact Effective Population Size (*N_e_*) and Historical Changes in *N*_*e*_?

**FIGURE 3:**
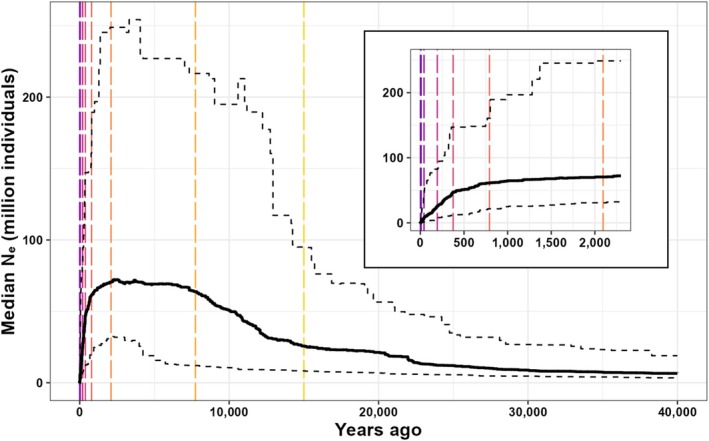
The reconstructed demographic history of the entire population sampled, showing effective population size (*N*_*e*_) over time as inferred by demographic modeling. Note that the *x*‐axis is shown as going back in time, as this is the direction of the reconstruction. The mutation rate was set to 1.8 × 10^−8^ per site per generation with a generation time of 2 years. The inset restricts the range to 2,300 ya. The black solid line represents the median *N*_*e*_ while the black dashed lines represent the 2.5 and 97.5 percentile estimations. Colored vertical lines represent the starts of periods with distinct rates of change.

The reconstructed trends in *N*_*e*_ over time did not differ between urban and rural habitats, but showed changes consistent with longer‐term anthropogenic disturbance (Figure 3, Figure S19). The breakpoint analysis for the entire population identified nine periods with distinct rates of change in *N*_*e*_ (Figure S20, Table S24). The results indicate a gradual increase in *N*_*e*_ from ~22,000 years ago (ya) through the end of the last glacial maximum (~19,000 years ago; Clark et al. 2009), and a weaker increase from ~7,742 ya to 2,094 ya. *N*_*e*_ remained relatively high during this period, then slowly declined until a sharp decrease approximately 792 ya that intensified around 376 ya. Thereafter, the rate of loss of *N*_*e*_ increased at each breakpoint (estimated at 194 ya, 42 ya, 9 ya, and 2 ya).

In both habitats and for the entire population, *N*_*e*_ declined > 99% from ~792 ya until present. When urbanization was categorized based on Distance from the City Center, *N*_*e*_ in the urban and rural groups declined from ~67 million to ~59,000 (CI: 8,000‐321,000), and from ~59 million to ~109,000 (CI: 25,000‐588,000), respectively. The *N*_*e*_ of the entire population declined from ~61 million to ~18,000 (CI: 5,000‐94,000). Simulations with varying mutation rates and a one‐year generation time showed similar recent sharp declines, though the estimated time of the decline varied (~500–2,500 ya; Figures S21–S23). Reconstructed trends in *N*_*e*_ were qualitatively identical when urbanization was classified based on Urbanization Score.

## 4 Discussion

Here, we sought to understand how urbanization impacted genetic diversity, demographic change, genetic differentiation, and spatial genetic structure in common milkweed in the GTA. We also asked whether these effects were consistent with the urban facilitation model, the urban fragmentation model, or a null model. Overall, we found little evidence that urbanization influenced the distribution of neutral genetic variation in *A. syriaca* . Specifically, genetic diversity and patterns of genetic differentiation did not differ between urban and rural habitats on average, with most differentiation occurring among individuals within sampling sites. Likewise, we found that the sampled plants originated from a single genetic population and there was little spatial genetic structure (Q1). Historical changes in effective population size were also consistent among urban and rural habitats, showing a moderate decline from ~2,094 ya until the beginning of a severe decline ~792 ya (Q2). Taken together, these results align best with the pre‐urbanization null model, which suggests that long‐term anthropogenic disturbances have had a prominent effect on demographic processes through time. These results could indicate that *A. syriaca* is similarly affected by human‐impacted environments (e.g., urban and rural habitats), in contrast to environments that are not heavily altered by humans (i.e., “natural” habitats).

### 4.1 Support for the Pre‐Urbanization Null Model

At the outset, several findings from our study align with expectations of the classic null model, which predicts negligible differences in genetic diversity and spatial genetic structure between urban and nonurban areas. For instance, when urban and rural habitats were compared, we found similar measures of genetic diversity, Tajima's D, present estimates of *N*_*e*_, qualitatively consistent reconstructed *N*_*e*_ histories, and little genetic differentiation among sampling sites between these habitats. Two more main results coincide with these findings: support for the existence of a single genetic population with continuous genetic variation and little evidence of spatial genetic structure. However, demographic modeling and breakpoint analysis showed a sharp drop in effective population size ~792 ya that coincides more so with human landscape alteration rather than modern urbanization. This finding is essential for realigning our results from the classic null model to the pre‐urbanization null model. Further evidence suggesting that human landscape alteration before and after European settlement, rather than modern urbanization, has driven a large drop in effective population size that has accelerated through time is discussed in greater detail in the following section.

Perhaps due to the relative novelty of applying demographic modeling analysis to urban evolutionary biology, few studies have involved the analyses essential for evaluating, let alone shown support for, the pre‐urbanization null model (but see Moran et al. 2023 and Wei et al. 2021). However, our results regarding genetic diversity, spatial genetic structure, and modern *N_e_* are comparable to a wider array of studies—only some of which show results similar to ours. We found little evidence that urbanization impacted genetic diversity in *A. syriaca* , a trend observed in other plants (Korpelainen et al. 2012; Caizergues et al. 2024), birds (Schmidt et al. 2020), and amphibians (Schmidt et al. 2022). However, recent meta‐analyses showed signals of urbanization decreasing genetic diversity within populations of mammals (Schmidt et al. 2020) and diverse taxa (Miles et al. 2019)—an effect mirrored in the plants *Impatiens capensis* (Rivkin and Johnson 2022) and *Linaria vulgaris* (Bartlewicz et al. 2015). There was low genetic differentiation between urban and rural groups, which has been observed in multiple plant species (Culley et al. 2007; Bartlewicz et al. 2015; Caizergues et al. 2024; but see Korpelainen et al. 2012 and Johnson et al. 2018) and birds (Schmidt et al. 2020). Lastly, while we found relatively neutral effects on modern *N*_*e*_ consistent with a study of the plant *Trifolium repens* (Caizergues et al. 2024), urbanization is generally predicted to decrease *N*_*e*_ (Miles et al. 2019; Ellwanger et al. 2022). We anticipate better understanding the generality of our findings as more studies investigating demographic history in urban environments are published.

Beyond urban environments, our results are generally consistent with studies of *A. syriaca* . For instance, we found relatively low genetic diversity, which is common among populations of *Asclepias* spp. (Edwards and Wyatt 1994; Sussman 2017), including *A. syriaca* , even across its North American range (Boyle et al. 2023). The lack of population genetic structure also aligns with other research in this genus showing low genetic differentiation among *A. speciosa* populations sampled across a 1,500 km gradient in the Northwestern US (Sussman 2017) and *A. syriaca* populations sampled across the species' North American range (Boyle et al. 2023). Boyle et al. (2023) also inferred the presence of one nearly panmictic population. Additionally, our AMOVA results closely align with those from a study of *A. syriaca* populations across its native range, which also showed that most genetic variation was within sampling sites (~90%) and the rest was between sampling sites (Agrawal et al. 2015).

There are several other factors that could prevent urbanization from exerting strong impacts on gene flow and genetic drift in common milkweed located in the GTA. First, the species' clonal and perennial nature may have prevented evolution from occurring within the approximately 150 years since the Toronto human population has exceeded 50,000 (Canada Department of Agriculture 1873). The already high seed dispersal distance potential may be elevated in urban areas due to increased air turbulence and temperatures (Morse and Schmitt 1985; Sacchi 1987; Kuparinen et al. 2009), and high pollen dispersal is likely driven by a wide variety of potential pollinators, with the ability of several to thrive in urban areas specifically (MacIvor et al. 2017; Baker and Potter 2018; Breitbart et al. 2023b). The recent rise of urban “pollinator gardens”, which often contain common milkweed, likely supports and attracts viable pollinators, and the resultant distribution of introduced milkweed genotypes across the landscape could erode natural population genetic structure that has evolved over millennia. More broadly, the similarly low genetic diversities of urban and rural groups, which is corroborated by our detection of several recent and strong declines in *N*_*e*_, also prompts questions about the capacity for additional environmental pressures like urbanization and even the development of rural environments to substantially impact genetic diversity if it is exceptionally low at the outset. While these factors may reinforce patterns associated with the classic null model, temporal contextualization through demographic analysis is essential for a more comprehensive interpretation of these results.

### 4.2 Genomic Signatures of Pre‐Urbanization Human Landscape Change

Our demographic modeling and breakpoint analysis suggested an expansion from ~22,000 ya until ~2,094 ya, followed by a gradual decline and then a sharp drop starting ~792 ya. Alternative models that assumed different mutation rates also replicated the sharp downturn and placed it around the same time (between 500–2,500 ya). We acknowledge that these timelines are estimates with uncertainty and recommend that these dates be interpreted with caution; the exact timing should not be overinterpreted. Despite this caveat, this finding is essential for aligning our results with the pre‐urbanization null model and suggests that human landscape alteration before and after European settlement, rather than modern urbanization, has driven a large drop in effective population size that has accelerated through time.

Our demographic history reconstructions contrast with those of a study investigating demographic changes in *A. syriaca* across its native range (Boyle et al. 2023). Boyle et al. (2023) detected similar increases in *N*_*e*_ after the last glacial maximum but did not detect a subsequent bottleneck across the species' “broad” range (i.e., across the North American habitat) or “core” range (i.e., across the eastern portion of the broad range). This discrepancy may originate from the differences in sampling location, breadth, or density. For instance, our study incorporated approximately 2,800 SNPs from 256 plants from one urban area whereas Boyle et al. (2023) incorporated approximately 900 SNPs from ~50–100 total genotyped plants covering the species' broad or core geographic ranges, depending on the dataset. A higher‐density sampling design (i.e., more SNPs within more individuals, sampled from a small geographic area) could have increased our power in estimating *N*_*e*_ and detecting demographic changes reflective of a specific geographic area (Marandel et al. 2020). Resultantly, more power may have helped increase the accuracy of *N*_*e*_ estimation, despite complications from this species' life history traits including clonality, hermaphroditism, overlapping generations, and an unknown mutation rate (Nunney 1993). Nonetheless, our demographic model appears to detect the outcome of intense landscape‐altering periods that were local to southern Ontario.

Over time, the landscapes in and near the GTA were transformed by Indigenous practices and European settlement. Simultaneously, *N*_*e*_ in the GTA initially increased but then began a long decline during a time of Indigenous settlement that accelerated quickly within the past century. Indigenous Peoples have lived on the land comprising the modern‐day GTA since at least the last glacial maximum (Johnson 2013), when *N*_*e*_ of the GTA population was gradually increasing. By ~2,094 ya, *N*_*e*_ had begun to decrease, preceding early corn cultivation along the Grand River in Southern Ontario (~1,500 ya) (Riley 2013). *N*_*e*_ decreased more quickly after ~792 ya, shortly before paleobotanical evidence from Halton, Ontario, suggests the Iroquois cultivated diverse crops in the area (i.e., for multiple periods during ~500–700 ya) (McCarthy et al. 2023). Indigenous agriculture and settlement directly impacted about 5% of the land in Ontario south of the Canadian Shield, though their true influence was around 3–4x higher because of active forest management (e.g., controlled fires, coppicing) (Riley 2013).

Studies in urban ecology and evolution rarely incorporate temporal dynamics into their frameworks (Ramalho and Hobbs 2012; Moll et al. 2019). Our results highlight the importance of considering the eco‐evolutionary impact of land‐use history before urbanization. Specifically, the dramatic decline in reconstructed *N*_*e*_ over the last 2,000 years cannot be explained by climate alone (Osman et al. 2021). Indeed, several estimated breakpoints align well with changes in the land‐use history of the Great Lakes region. The arrival of European colonists to North America launched a series of events that transformed the landscape. By another breakpoint at 376 ya, Eastern North America had become an “epidemic region” due to the introduction of diseases that severely reduced Indigenous populations (Riley 2013) and thus reduced their impact on the land, starting a period of rewilding. The rate of change in *N*_*e*_ accelerated again at 194 ya, around when vast swaths of southern Ontario were being cleared for logging, agriculture, and settlement (Riley 2013; Aleksa 2022), and by ~100 ya, an estimated 94% of upland woodlands had been converted to farmland (Riley 2013). Simultaneously, settlers prized prairies for their ease in conversion to agricultural areas; only traces are left in Eastern North America (Riley 2013). Since ~100 ya, multiple efforts in Southern Ontario and the GTA have encouraged reforestation (Riley 2013; TRCA 2017; Aleksa 2022) and planting milkweed, specifically, though growing urban expansion may have intensified a progressively rapid decrease in *N*_*e*_ through the last breakpoints at 42, 9, and 2 ya. While our demographic model's steep decline coincides with the widespread loss of woodlands and prairie, the latter of which is prime habitat for common milkweed, others concluded that the reduction of these environments facilitated the growth and spread of common milkweed in North America (Brower 1995; Malcolm 2018). Sampling other common milkweed populations in the Great Lakes region with similar depth and breadth as in this study, and reconstructing their demographic histories, could clarify the geographical generality of our results and potentially resolve the discrepancy with other studies.

## 5 Limitations

There are multiple limitations of this study. First, as our sampling occurred exclusively within the GTA, we cannot extrapolate our results beyond this single city. Repeating this study in cities that vary in age, climate, developmental history, and which occur across the geographic range of *A. syriaca* would provide key information about the specificity of our results to the GTA. Secondly, we cannot confidently identify the factors that determined our demographic model history reconstruction. While it is sensible to compare the natural history of our study area's environs with our results, we acknowledge that our models likely reflect consequences of other concurrent events such as biotic changes (e.g., the introduction or loss of species, including pathogens) or abiotic changes (e.g., alterations to the climate or geological landscape). Lastly, as stated previously, our demographic modeling was shaped by multiple assumptions, some of which were unknown (e.g., mutation rate), while others could not be met (e.g., nonoverlapping generations). We were able to verify the robustness of our results by performing a sensitivity analysis, but cannot definitively prove the accuracy of our results. Repeating the modeling upon identification of these currently unknown parameters could help overcome this limitation, but specific life history traits will likely continue to complicate efforts until the development of a more powerful tool. Despite these limitations, our study still provides evidence that anthropogenic activity before urbanization has substantially influenced non‐adaptive evolution in common milkweed growing in the GTA.

## 6 Conclusion

Little is known about how urban landscapes influence non‐adaptive evolution in populations, especially in plants. Here, we show evidence that urbanization has not substantially influenced gene flow or genetic drift in common milkweed within the GTA. Furthermore, a sharp decline in effective population size prior to urbanization may be concomitant with a considerable reduction in genomic diversity. The legacy of this decline, which coincides with precontact Indigenous settlement and European settler colonialism in this region, may have buffered against further changes in genetic diversity and differentiation due to urbanization in this population. Resultantly, there may be few impacts of urbanization on the eco‐evolutionary dynamics between this population of common milkweed and its pollinators. These results emphasize the complexity of socio‐ecological dynamics and underscore the importance of evaluating scientific findings within local and historical contexts (Des Roches et al. 2021; Schell et al. 2020; Moran et al. 2023). Thus, we advocate that future studies account for ecological histories to thoroughly contextualize patterns of genetic diversity and differentiation in urban landscapes. We also propose that researchers study non‐adaptive evolution across various species in the same urban area, and the same species in multiple urban areas, to clarify how socio‐ecological histories of different regions interact with diverse life histories and ecological niches. Examining the feedbacks between adaptive and non‐adaptive evolutionary processes could also shed light on how population genetic parameters are shaped. Overall, embracing intersectionality is critical for accurately identifying the processes responsible for generating the genomic patterns we observe in urban environments.

## Author Contributions

**Sophie T. Breitbart:** conceptualization (equal), data curation (lead), formal analysis (equal), funding acquisition (supporting), investigation (lead), methodology (equal), project administration (equal), software (lead), visualization (lead), writing – original draft (lead), writing – review and editing (equal). **Marc T. J. Johnson:** conceptualization (equal), data curation (supporting), formal analysis (equal), funding acquisition (lead), investigation (supporting), methodology (equal), project administration (equal), resources (lead), supervision (equal), visualization (supporting), writing – review and editing (equal). **Helene H. Wagner:** conceptualization (equal), data curation (supporting), formal analysis (equal), funding acquisition (lead), investigation (supporting), methodology (equal), project administration (equal), resources (lead), supervision (equal), visualization (supporting), writing – review and editing (equal).

## Conflicts of Interest

The authors declare no conflicts of interest.

## Supporting information


Appendix S1.

## Data Availability

Data and code are archived on Zenodo (https://doi.org/10.5281/zenodo.14911290). DNA sequence data were deposited in the NCBI Short Read Archive (SRA) under BioProject PRJNA1127624.
