# Supplementary material for: Anthropogenic Landscape Alteration, but Not Urbanization, Influences Non‐Adaptive Evolution in Common Milkweed ( Asclepias syriaca L.)
Source: Ecol Evol. 2025 Apr 18;15(4):e71250. doi: 10.1002/ece3.71250 (PMC12008043; doi:10.1002/ece3.71250)
Supplement: Supplementary file 1 [file ECE3-15-e71250-s001.odt]

Supplemental Information for:

Anthropogenic landscape alteration, but not urbanization, influences non-adaptive evolution in common milkweed (Asclepias syriaca L.)

Sophie T. Breitbart, Marc T.J. Johnson, Helene H. Wagner

Table of Contents

- Supplementary text S1

- Supplementary figures S1-S23

- Supplementary tables S1-S24

- Supplementary references

Supplementary Text S1

Methods

We extracted DNA from leaf tissue with the Qiagen DNeasy Plant Kit as per the manufacturer’s instructions with the following modifications: Adding 500 μl AP1 buffer instead of 400 μl (+ 4 μl RNAse); incubating samples for 15 minutes at 65ºC and mixing 3 times during incubation by inverting the tube; adding 163 μl P3 and mixing by inverting tubes 5-6 times, then placing the tube on ice for 5 minutes and centrifuging for 15 minutes at 14000 rpm; adding 1.5x AW1 based on the recovered lysate volume, which was >450 μl (typically 500-550 μl); eluting with 35 μl AE buffer for first elution and 25 μl for the second elution, allowing at least 10 minutes each time to improve yield.

Question 1: How does urbanization influence genetic diversity and population genetic structure?

First, we used principal component analysis (PCA) to examine genomic structure through ordination. We generated eigenvector and eigenvalue data with PLINK, then visualized the PCA in R. We also used a spatially-explicit method, Discriminant Analysis of Principal Components (DAPC) (Jombart et al. 2010), to maximize differences among potential groups. We used the package adegenet v2.1.10 (Jombart 2008) to calculate the Bayesian information criteria (BIC) for each number of possible clusters and examine cluster membership, retaining all 256 PCs.

We used permutational analysis of variance (PERMANOVA) (Anderson 2001) to determine whether genetic distances differed between the urban and rural habitats. We treated a distance matrix consisting of the PCA’s PC axes as a response variable in the following models, which were fitted with the “adonis2” function from the package vegan v2.6.4 (Oksanen et al. 2019):

Response variable ~ Distance from the City Center OR Urbanization Score + Error        (Eq. S1)

In these models, Distance from the City Center and Urbanization Score are categorical fixed effects, signifying whether an individual belonged to the urban or rural habitat. We controlled for individuals belonging to the same sampling site and ran models with 1000 permutations.

We used permutational multivariate analysis of dispersion (PERMDISP) to test whether the variance in genetic distances differed between the urban and rural habitats with the “betadisper” function from vegan.

To test for positive spatial autocorrelation consistent with isolation-by-distance (IBD) by creating Mantel correlograms, we created correlograms for all genotyped individuals from the urban and rural groups using Euclidean distances of allele frequencies with 1000 permutations, progressive Holm corrections (Holm 1979) for multiple testing (with alpha = 0.05 for the first distance class), and a two-sided alternative (the sole option). Distance class definition varied between urban and rural habitats to reflect typical distances between sampling sites: urban models used 2.5 km and 10 km distance lags while rural models used 10 km lags, because apart from the sites sampled along the transect, most urban and rural sites were sampled using a 2.5 km or a 10 km grid, respectively. For the memgene analysis, we set alpha to 0.05, measured genetic distances among individuals as the proportion of shared alleles, and used 1000 permutations for the forward selection of MEM eigenvectors.

Question 2: How does historical anthropogenic change impact effective population size (Ne) and historical changes in Ne?

Since the mutation rate for common milkweed is unpublished, we modelled effective population size with a range of mutation rates based on values from species with similar taxonomy and/or lifespans. We ran models with the following mutation rates: 1.0 x 10-8, 1.2 x 10-8 (Homo sapiens, modern human), 1.8 x 10-8 (Trifolium repens, white clover), and 2.6 x 10-8 (Picea stichensis, Sitka spruce) (Hanlon et al. 2019).

Results

The first two principal axes of the PCA explained 6.21% and 6.02% of the total genetic variation, respectively, and formed one rough cluster (Figure S8). DAPC identified 1 population (BIC = 1253.28) (Figure S9). The conStruct model with K = 1 was considered the best model since the K = 2 model did not have higher predictive accuracy and one layer in the K = 2 model contributed only 5% covariance to the overall model (Tables S9-10).

When the analyses were restricted to one individual per sampling site and urbanization was based on Urbanization Score, the Mantel correlograms revealed positive spatial autocorrelation (PSA) for the urban group only, with the 10 km distance lag at the 15 km distance class (Figure S12, Table S17). The memgene analysis identified zero significant Moran eigenvector maps (MEM) for the urban group and four significant MEM for the rural group (Figure S13, Table S18).

When all genotyped individuals were included in the analysis and urbanization was based on Distance from the City Center, the Mantel correlograms revealed PSA for the rural group only, at the shortest (5 km) distance class (Figure S14, Table S19). There was no PSA when urbanization was classified based on Urbanization Score (Figure S15, Table S20). The memgene analysis identified zero significant MEM for urban and rural groups with both metrics of urbanization, signifying no detectable spatial structure in the genomic data for any group.

Analyses for the entire population supported the conStruct results suggesting the presence of continuous genetic variation, but only when all genotyped individuals were included. When the analyses were restricted to one individual per sampling site, the Mantel correlogram did not detect PSA and the memgene analysis identified zero significant MEM (Figure S16, Table S21). When all genotyped individuals were included, the Mantel correlogram showed PSA at the shortest distance class of 2.5 km and the memgene analysis identified 20 significant MEM (Figures S17-18, Tables S22-23).

A graph of different colored bars

Description automatically generated with medium confidence

Figure S1

Barplots showing the number of sampling sites with 1-5 genotyped individuals, separately for urban and rural habitats when urbanization was classified based on A) Distance to the City Center or B) Urbanization Score.

A graph of a distance from the city center

Description automatically generated

Figure S2

The correlation between both metrics of urbanization: Distance from the City Center and Urbanization Score. A regression line with a 95% confidence envelope and points representing sampling sites are shown for the general linear model.

A map with red and blue dots

Description automatically generated

Figure S3

Map of 124 common milkweed sampling sites throughout the Greater Toronto Area. Red triangles (n = 64) and blue circles (n = 60) indicate classification as “Rural” and “Urban”, respectively, based on Urbanization Score (as opposed to Distance from the City Center, show in Figure 2). The ESRI world imagery basemap shows urban and suburban areas in light gray, non-urban agricultural and forested areas in green, and Lake Ontario in blue. Tiles © Esri — Source: Esri, i-cubed, USDA, USGS, AEX, GeoEye, Getmapping, Aerogrid, IGN, IGP, UPR-EGP, and the GIS User Community.

A comparison of a graph

Description automatically generated with medium confidence

Figure S4

The effect of urbanization on π when urbanization was classified based on A) Distance from the City Center and B) Urbanization Score. Regression lines represent predicted values with a 95% confidence interval and points represent sampling site-level values.

A graph of a number of points

Description automatically generated with medium confidence

Figure S5

The effect of urbanization on FIS when urbanization was classified based on A) Distance from the City Center and B) Urbanization Score. Regression lines represent predicted values with a 95% confidence interval and points represent sampling site-level values.

A graph showing the number of different numbers

Description automatically generated with medium confidence

Figure S6

Tajima’s D across 100 kbp windows for the A) entire population, B) urban habitat when urbanization was classified based on Distance from the City Center, C) urban habitat when urbanization was classified based on Urbanization Score, D) rural habitat when urbanization was classified based on Distance from the City Center, E) rural habitat when urbanization was classified based on Urbanization Score. Tajima’s D across the genome is shown in the center.

A diagram of a graph

Description automatically generated with medium confidence

Figure S7

A) Cross-validation results for data simulated under K = 1–5 (the number of hypothesized genetic populations), comparing the spatial (empty circles) and nonspatial (filled circles) conStruct models. Higher predictive accuracy values indicate better model performance. B) Layer contributions for spatial models simulated under K = 1–5. The model with K = 1 was considered the best model since the K = 2 model did not have higher predictive accuracy and one layer in the K = 2 model contributed only 5% covariance to the overall model.

A graph showing a number of cities

Description automatically generated with medium confidence

Figure S8

Principal component analysis (PCA) for 256 samples when urbanization was classified based on A) Distance from the City Center and B) Urbanization Score. PCs explaining the most variation are on the x and y axes, respectively.

A comparison of a graph

Description automatically generated

Figure S9

A) Cumulative variance explained by the eigenvalues of 1-256 PCs. B) Bayesian information criterion (BIC) for 1-25 clusters.

Figure S10

Mantel correlograms for A) urban individuals with 2.5 km distance lags, B) urban individuals with 10 km distance lags, and C) rural individuals with 10 km distance lags, when urbanization was classified based on Distance from the City Center and the analysis was restricted to one individual per sampling site. The x and y axes show Euclidean distances and Mantel r, respectively, and white circles indicate non-significant spatial autocorrelation for a given distance class.

A screenshot of a computer generated image

Description automatically generated

Figure S11

Results from memgene analysis for rural individuals when urbanization was classified based on Distance from the City Center and analysis was restricted to one individual per sampling site. Shown are memgene variables A) 1 and B) 2, which explained significant spatial structure in the genomic data. The ESRI world imagery basemap shows urban and suburban areas in light gray, non-urban agricultural and forested areas in green, and Lake Ontario in blue (ESRI World Imagery Basemap 2023). Tiles © Esri — Source: Esri, i-cubed, USDA, USGS, AEX, GeoEye, Getmapping, Aerogrid, IGN, IGP, UPR-EGP, and the GIS User Community.

Figure S12

Mantel correlograms for A) urban individuals with 2.5 km distance lags, B) urban individuals with 10 km distance lags, and C) rural individuals with 10 km distance lags, when urbanization was classified based on Urbanization Score and analysis was restricted to one individual per sampling site. The x and y axes show Euclidean distances and Mantel r, respectively, and black squares indicate significant spatial autocorrelation for a given distance class.

A collage of images of different colors

Description automatically generated

Figure S13

Results from memgene analysis for rural individuals when urbanization was classified based on Urbanization Score and analysis was restricted to one individual per sampling site. Shown are memgene variables A) 1, B) 2, C) 3, and D) 4, which explained significant spatial structure in the genomic data. The ESRI world imagery basemap shows urban and suburban areas in light gray, non-urban agricultural and forested areas in green, and Lake Ontario in blue (ESRI World Imagery Basemap 2023). Tiles © Esri — Source: Esri, i-cubed, USDA, USGS, AEX, GeoEye, Getmapping, Aerogrid, IGN, IGP, UPR-EGP, and the GIS User Community.

Figure S14

Mantel correlograms for A) urban individuals with 2.5 km distance lags, B) urban individuals with 10 km distance lags, and C) rural individuals with 10 km distance lags, when urbanization was classified based on Distance from the City Center and all genotyped individuals were included. The x and y axes show Euclidean distances and Mantel r, respectively, and black squares indicate significant spatial autocorrelation for a given distance class.

Figure S15

Mantel correlograms for A) urban individuals with 2.5 km distance lags, B) urban individuals with 10 km distance lags, and C) rural individuals with 10 km distance lags, when urbanization was classified based on Urbanization Score and all genotyped individuals were included. The x and y axes show Euclidean distances and Mantel r, respectively, and white circles indicate non-significant spatial autocorrelation for a given distance class.

Figure S16

Mantel correlograms for the entire population and the analysis was restricted to one individual per sampling site with A) 2.5 km and B) 10 km distance lags. The x and y axes show Euclidean distances and Mantel r, respectively, and white circles indicate non-significant spatial autocorrelation for a given distance class.

Figure S17

Mantel correlograms for the entire population and all individuals were included with A) 2.5 km and B) 10 km distance lags. The x and y axes show Euclidean distances and Mantel r, respectively, and black squares indicate significant spatial autocorrelation for a given distance class.

A group of images of different colors

Description automatically generated with medium confidence

Figure S18

Results from memgene analysis when all genotyped individuals were included. Shown are memgene variables A) 1, B) 2, C) 3, and D) 4, which explained the most significant spatial structure in the genomic data of all 20 memgene variables. The ESRI world imagery basemap shows urban and suburban areas in light gray, non-urban agricultural and forested areas in green, and Lake Ontario in blue (ESRI World Imagery Basemap 2023). Tiles © Esri — Source: Esri, i-cubed, USDA, USGS, AEX, GeoEye, Getmapping, Aerogrid, IGN, IGP, UPR-EGP, and the GIS User Community.

Figure S19

The reconstructed demographic history showing effective population size (Ne) over time as inferred by demographic modeling for urban and rural habitats when urbanization was classified based on A) Distance from the City Center and B) Urbanization Score. Note that the x-axis is shown as going back in time, as this is the direction of the reconstruction. The mutation rate was set to 1.8 x 10-8 per site per generation with a generation time of two years. The solid lines represent the median Ne while the dashed lines represent the 2.5 and 97.5 percentile estimations (Rural is grey; Urban is black). The inset restricts the range to 2,300 ya. Colored vertical lines represent the starts of periods with distinct rates of change for the main model (i.e., mutation rate = 1.8 x 10-8 per site per generation and generation time = 2 years).

A						     B

A graph of a graph of a graph

Description automatically generated with medium confidence

Figure S20

Linear and piecewise regression models of the reconstructed demographic modelling data for the entire population sampled with the main parameters (i.e., mutation rate = 1.8 x 10-8 per site per generation and generation time = 2 years) for the following years before present: A) 0-15,000 and B) 0-1,000 (an inset of A).

A graph of different colored lines

Description automatically generated with medium confidence

Figure S21

The reconstructed demographic history of the entire population sampled, showing effective population size (Ne) over time as inferred by demographic modeling, with the following mutation rates: A) 1.0 x 10-8 , B) 1.2 x 10-8 , C) 1.8 x 10-8 , D) 2.6 x 10-8 per site per generation with a generation time of two years. Note that the x-axis is shown as going back in time, as this is the direction of the reconstruction. The black solid line represents the median Ne while the black dashed lines represent the 2.5 and 97.5 percentile estimations. The inset restricts the range to 2,300 ya. Colored vertical lines represent the starts of periods with distinct rates of change for the main model (i.e., mutation rate = 1.8 x 10-8 per site per generation and generation time = 2 years).

Figure S22

The reconstructed demographic history showing effective population size (Ne) over time as inferred by demographic modeling for A) the entire population, and urban and rural habitats when urbanization was classified based on B) Distance from the City Center and C) Urbanization Score. Note that the x-axis is shown as going back in time, as this is the direction of the reconstruction. The mutation rate was set to 1.8 x 10-8 per site per generation with a generation time of one year (vs. two years in Figures 3 and S16). The solid lines represent the median Ne while the dashed lines represent the 2.5 and 97.5 percentile estimations (panels B & C: Rural is grey; Urban is black). Colored vertical lines represent the starts of periods with distinct rates of change for the main model (i.e., mutation rate = 1.8 x 10-8 per site per generation and generation time = 2 years).

A graph of a number of different colored lines

Description automatically generated with medium confidence

Figure S23

The reconstructed demographic history of the entire population sampled, showing effective population size (Ne) over time as inferred by demographic modeling, with the following mutation rates: A) 1.0 x 10-8 , B) 1.2 x 10-8 , C) 1.8 x 10-8 , D) 2.6 x 10-8 per site per generation with a generation time of one year (vs. two years in Figures 3 and S9). Note that the x-axis is shown as going back in time, as this is the direction of the reconstruction. The black solid line represents the median Ne while the black dashed lines represent the 2.5 and 97.5 percentile estimations. The inset restricts the range to 2,300 ya. Colored vertical lines represent the starts of periods with distinct rates of change for the main model (i.e., mutation rate = 1.8 x 10-8 per site per generation and generation time = 2 years).

Table S1

PLINK results showing pairs of genotyped individuals that contained at least 25% identity by descent. Shown are the individuals and the associated sampling sites, the proportion of identity by descent, and the distance between sampling sites. Samples labelled “Individual 1” were removed from the dataset. Since sampling site MWI037 contained a single individual (3A_E7_sorted), withdrawing this sample removed the site from the analysis. 

| Individual 1 | Sampling Site 1 | Individual 2 | Sampling Site 2 | Proportion Identity by Descent | Distance between Sites (km) |
| --- | --- | --- | --- | --- | --- |
| 1.2_1A6_sorted | MW036 | 1.2_1F1_sorted | MW046 | 0.3928 | 7.6683 |
| 1.2_1E9_sorted | MW004 | 3A_G3_sorted | MWI004 | 0.2791 | 24.2170 |
| 1.2_1F6_sorted | MW017 | 1.2_2F8_sorted | MW017 | 0.4325 | 0 |
| 1.2_2D3_sorted | MW067 | 3A_F12_sorted | MWI080 | 0.5028 | 16.0546 |
| 3A_E7_sorted | MWI037 | 3A_E8_sorted | MWI045 | 0.7352 | 66.0310 |

Table S2

Stacks summary statistics for variant positions within dataset #1. The following parameters are shown for each sampling site: private alleles, mean individuals per locus, mean observed (Ho) and expected (He) heterozygosity, mean nucleotide diversity (π), and mean inbreeding coefficient (FIS).

| Sampling Site | Private | Individuals | Ho | He | π | FIS |
| --- | --- | --- | --- | --- | --- | --- |
| MW067 | 0 | 3.22202 | 0.07878 | 0.07617 | 0.09272 | 0.02431 |
| MW079 | 1 | 4.03362 | 0.08384 | 0.09002 | 0.104 | 0.04208 |
| MW040 | 1 | 3.18768 | 0.08939 | 0.0874 | 0.10537 | 0.02903 |
| MW047 | 0 | 4.02844 | 0.07632 | 0.08269 | 0.0954 | 0.03862 |
| MW002 | 2 | 4.20757 | 0.08247 | 0.09385 | 0.10691 | 0.05134 |
| MW012 | 0 | 4.1983 | 0.08571 | 0.08537 | 0.09836 | 0.02525 |
| MW013 | 1 | 2.44172 | 0.08694 | 0.07419 | 0.09503 | 0.01266 |
| MW018 | 0 | 3.32515 | 0.08917 | 0.08599 | 0.10171 | 0.02292 |
| MW030 | 1 | 3.24258 | 0.08065 | 0.08084 | 0.09682 | 0.0301 |
| MW020 | 0 | 1 | 0.07856 | 0.03928 | 0.07856 | 0 |
| MW028 | 0 | 1.68877 | 0.09134 | 0.07026 | 0.10117 | 0.01473 |
| MW019 | 0 | 3.36064 | 0.08038 | 0.07567 | 0.09113 | 0.02023 |
| MW004 | 0 | 3.40205 | 0.07917 | 0.08328 | 0.09872 | 0.03648 |
| MW021 | 1 | 3.42142 | 0.07283 | 0.07393 | 0.08577 | 0.02427 |
| MW017 | 0 | 3.28684 | 0.07966 | 0.07545 | 0.08929 | 0.01757 |
| MW051 | 0 | 1.81621 | 0.08144 | 0.06117 | 0.08657 | 0.0077 |
| MW007 | 0 | 3.61934 | 0.06999 | 0.08421 | 0.0979 | 0.05569 |
| MW056 | 0 | 2.30248 | 0.07528 | 0.06745 | 0.08676 | 0.01876 |
| MW057 | 1 | 4.13277 | 0.08392 | 0.08894 | 0.10218 | 0.03907 |
| MW027 | 0 | 2.56268 | 0.08567 | 0.07298 | 0.09213 | 0.01026 |
| MW049 | 2 | 4.21565 | 0.08216 | 0.08078 | 0.09273 | 0.02167 |
| MW077 | 3 | 3.72747 | 0.08112 | 0.08005 | 0.09423 | 0.02635 |
| MW039 | 0 | 4.03325 | 0.07758 | 0.08928 | 0.10267 | 0.05154 |
| MW001 | 2 | 3.6688 | 0.079 | 0.08131 | 0.09616 | 0.03435 |
| MW006 | 0 | 4.02654 | 0.08087 | 0.0907 | 0.10502 | 0.04954 |
| MW072 | 1 | 3.95623 | 0.07995 | 0.08461 | 0.09774 | 0.03671 |
| MW023 | 0 | 1 | 0.08795 | 0.04398 | 0.08795 | 0 |
| MW036 | 2 | 3.45387 | 0.08052 | 0.0831 | 0.09795 | 0.03391 |
| MW062 | 0 | 3.83084 | 0.08137 | 0.08789 | 0.1016 | 0.03957 |
| MW045 | 0 | 1 | 0.08156 | 0.04078 | 0.08156 | 0 |
| MW016 | 0 | 1 | 0.08671 | 0.04335 | 0.08671 | 0 |
| MW046 | 0 | 3.71546 | 0.07722 | 0.08046 | 0.09332 | 0.03136 |
| MW061 | 0 | 2.6134 | 0.08504 | 0.07179 | 0.09144 | 0.01048 |
| MW068 | 0 | 3.78112 | 0.07604 | 0.07663 | 0.08965 | 0.02689 |
| MW029 | 0 | 2.35426 | 0.07526 | 0.07249 | 0.09285 | 0.0291 |
| MW014 | 0 | 4.0263 | 0.07557 | 0.08986 | 0.10288 | 0.05511 |
| MW042 | 0 | 1.81722 | 0.08132 | 0.05971 | 0.084 | 0.00403 |
| MW003 | 0 | 2.94519 | 0.06975 | 0.06596 | 0.08101 | 0.01856 |
| MW031 | 1 | 3.96288 | 0.078 | 0.09088 | 0.10437 | 0.05442 |
| MW015 | 0 | 2.94096 | 0.08182 | 0.08352 | 0.10215 | 0.03578 |
| MW009 | 0 | 1.72083 | 0.07674 | 0.06496 | 0.09024 | 0.02026 |
| MW011 | 1 | 3.14327 | 0.0743 | 0.07318 | 0.08861 | 0.02623 |
| MW064 | 1 | 2.52643 | 0.08097 | 0.07353 | 0.09192 | 0.01818 |
| MW069 | 0 | 1.83248 | 0.08248 | 0.06094 | 0.08578 | 0.00495 |
| MW035 | 0 | 3.13126 | 0.07731 | 0.07783 | 0.09335 | 0.02912 |
| MW008 | 0 | 2.35104 | 0.0852 | 0.07323 | 0.09387 | 0.01317 |
| MW005 | 1 | 4.15773 | 0.08274 | 0.09116 | 0.10417 | 0.04434 |
| MW074 | 0 | 2.22531 | 0.06935 | 0.06748 | 0.0867 | 0.02896 |
| MW041 | 0 | 1.74471 | 0.07625 | 0.06183 | 0.08652 | 0.0154 |
| MWI067 | 0 | 1 | 0.07733 | 0.03867 | 0.07733 | 0 |
| MWI075 | 0 | 1 | 0.08375 | 0.04187 | 0.08375 | 0 |
| MWI007 | 0 | 1 | 0.08657 | 0.04328 | 0.08657 | 0 |
| MWI016 | 0 | 1 | 0.0801 | 0.04005 | 0.0801 | 0 |
| MWI025 | 0 | 1 | 0.07409 | 0.03704 | 0.07409 | 0 |
| MWI033 | 0 | 1 | 0.06912 | 0.03456 | 0.06912 | 0 |
| MWI041 | 0 | 1 | 0.08148 | 0.04074 | 0.08148 | 0 |
| MWI049 | 0 | 1 | 0.09877 | 0.04939 | 0.09877 | 0 |
| MWI058 | 0 | 1 | 0.08865 | 0.04432 | 0.08865 | 0 |
| MWI068 | 0 | 1 | 0.07726 | 0.03863 | 0.07726 | 0 |
| MWI076 | 0 | 1 | 0.08402 | 0.04201 | 0.08402 | 0 |
| MW026 | 0 | 1 | 0.08299 | 0.0415 | 0.08299 | 0 |
| MWI008 | 0 | 1 | 0.08043 | 0.04021 | 0.08043 | 0 |
| MWI017 | 0 | 1 | 0.08613 | 0.04307 | 0.08613 | 0 |
| MWI026 | 0 | 1 | 0.07981 | 0.0399 | 0.07981 | 0 |
| MWI034 | 0 | 1 | 0.07979 | 0.03989 | 0.07979 | 0 |
| MWI042 | 0 | 1 | 0.09385 | 0.04692 | 0.09385 | 0 |
| MWI050 | 0 | 1 | 0.0936 | 0.0468 | 0.0936 | 0 |
| MWI059 | 0 | 1 | 0.07692 | 0.03846 | 0.07692 | 0 |
| MWI069 | 0 | 1 | 0.08606 | 0.04303 | 0.08606 | 0 |
| MWI077 | 0 | 1 | 0.08772 | 0.04386 | 0.08772 | 0 |
| MWI009 | 0 | 1 | 0.09695 | 0.04848 | 0.09695 | 0 |
| MWI018 | 0 | 1 | 0.1006 | 0.0503 | 0.1006 | 0 |
| MWI027 | 0 | 1 | 0.11057 | 0.05528 | 0.11057 | 0 |
| MWI035 | 0 | 1 | 0.08614 | 0.04307 | 0.08614 | 0 |
| MWI043 | 0 | 1 | 0.07635 | 0.03817 | 0.07635 | 0 |
| MWI051 | 0 | 1 | 0.08294 | 0.04147 | 0.08294 | 0 |
| MWI060 | 0 | 1 | 0.08159 | 0.0408 | 0.08159 | 0 |
| MWI070 | 0 | 1 | 0.07978 | 0.03989 | 0.07978 | 0 |
| MWI078 | 0 | 1 | 0.09305 | 0.04653 | 0.09305 | 0 |
| MWI010 | 0 | 1 | 0.08188 | 0.04094 | 0.08188 | 0 |
| MWI019 | 0 | 1 | 0.07518 | 0.03759 | 0.07518 | 0 |
| MWI028 | 0 | 1 | 0.08384 | 0.04192 | 0.08384 | 0 |
| MWI036 | 0 | 1 | 0.08903 | 0.04452 | 0.08903 | 0 |
| MWI044 | 0 | 1 | 0.08356 | 0.04178 | 0.08356 | 0 |
| MWI052 | 0 | 1 | 0.07188 | 0.03594 | 0.07188 | 0 |
| MWI061 | 0 | 1 | 0.12088 | 0.06044 | 0.12088 | 0 |
| MWI071 | 0 | 1 | 0.08085 | 0.04043 | 0.08085 | 0 |
| MWI079 | 0 | 1 | 0.0832 | 0.0416 | 0.0832 | 0 |
| MWI001 | 0 | 1 | 0.06959 | 0.0348 | 0.06959 | 0 |
| MWI011 | 0 | 1 | 0.0752 | 0.0376 | 0.0752 | 0 |
| MWI020 | 0 | 1 | 0.07655 | 0.03828 | 0.07655 | 0 |
| MWI029 | 0 | 1 | 0.08093 | 0.04046 | 0.08093 | 0 |
| MWI045 | 0 | 1 | 0.11266 | 0.05633 | 0.11266 | 0 |
| MWI053 | 0 | 1 | 0.11513 | 0.05756 | 0.11513 | 0 |
| MWI062 | 0 | 1 | 0.09499 | 0.04749 | 0.09499 | 0 |
| MWI072 | 0 | 1 | 0.09008 | 0.04504 | 0.09008 | 0 |
| MWI080 | 0 | 1 | 0.07158 | 0.03579 | 0.07158 | 0 |
| MWI003 | 0 | 1 | 0.08477 | 0.04239 | 0.08477 | 0 |
| MWI014 | 0 | 1 | 0.08551 | 0.04275 | 0.08551 | 0 |
| MWI022 | 0 | 1 | 0.08376 | 0.04188 | 0.08376 | 0 |
| MWI030 | 0 | 1 | 0.08305 | 0.04153 | 0.08305 | 0 |
| MWI038 | 0 | 1 | 0.08599 | 0.043 | 0.08599 | 0 |
| MWI046 | 0 | 1 | 0.09685 | 0.04843 | 0.09685 | 0 |
| MWI054 | 0 | 1 | 0.09986 | 0.04993 | 0.09986 | 0 |
| MWI063 | 0 | 1 | 0.0837 | 0.04185 | 0.0837 | 0 |
| MWI073 | 0 | 1 | 0.08603 | 0.04301 | 0.08603 | 0 |
| UTSC | 0 | 1 | 0.07809 | 0.03905 | 0.07809 | 0 |
| MWI004 | 0 | 1 | 0.09191 | 0.04596 | 0.09191 | 0 |
| MWI015 | 0 | 1 | 0.07918 | 0.03959 | 0.07918 | 0 |
| MWI023 | 0 | 1 | 0.08386 | 0.04193 | 0.08386 | 0 |
| MWI031 | 0 | 1 | 0.07946 | 0.03973 | 0.07946 | 0 |
| MWI039 | 0 | 1 | 0.08008 | 0.04004 | 0.08008 | 0 |
| MWI047 | 0 | 1 | 0.07558 | 0.03779 | 0.07558 | 0 |
| MWI055 | 0 | 1 | 0.07532 | 0.03766 | 0.07532 | 0 |
| MWI066 | 0 | 1 | 0.09492 | 0.04746 | 0.09492 | 0 |
| MWI074 | 0 | 1 | 0.08212 | 0.04106 | 0.08212 | 0 |
| MWI005 | 0 | 1 | 0.07893 | 0.03946 | 0.07893 | 0 |
| MWI024 | 0 | 1 | 0.09191 | 0.04596 | 0.09191 | 0 |
| MWI032 | 0 | 1 | 0.08145 | 0.04072 | 0.08145 | 0 |
| MWI040 | 0 | 1 | 0.08676 | 0.04338 | 0.08676 | 0 |
| MWI048 | 0 | 1 | 0.08744 | 0.04372 | 0.08744 | 0 |
| MWI056 | 0 | 1 | 0.09415 | 0.04708 | 0.09415 | 0 |

Table S3

Stacks summary statistics for all positions (variant and fixed) within dataset #1. The following parameters are shown for each sampling site: private alleles, total sites, variant sites, mean individuals per locus, mean observed (Ho) and expected (He) heterozygosity, mean nucleotide diversity (π), and mean inbreeding coefficient (FIS).

| Sampling Site | Private | Sites | Variant sites | Individuals | Ho | He | π | FIS |
| --- | --- | --- | --- | --- | --- | --- | --- | --- |
| MW067 | 0 | 349323 | 2779 | 3.39322 | 0.00063 | 0.00061 | 0.00074 | 0.00019 |
| MW079 | 1 | 351966 | 2826 | 4.46318 | 0.00067 | 0.00072 | 0.00084 | 0.00034 |
| MW040 | 1 | 349431 | 2808 | 3.3483 | 0.00072 | 0.0007 | 0.00085 | 0.00023 |
| MW047 | 0 | 352220 | 2813 | 4.39305 | 0.00061 | 0.00066 | 0.00076 | 0.00031 |
| MW002 | 2 | 352150 | 2828 | 4.46686 | 0.00066 | 0.00075 | 0.00086 | 0.00041 |
| MW012 | 0 | 351966 | 2824 | 4.41017 | 0.00069 | 0.00069 | 0.00079 | 0.0002 |
| MW013 | 1 | 349127 | 2728 | 2.61505 | 0.00068 | 0.00058 | 0.00074 | 0.0001 |
| MW018 | 0 | 351498 | 2811 | 3.48402 | 0.00071 | 0.00069 | 0.00081 | 0.00018 |
| MW030 | 1 | 349642 | 2795 | 3.42987 | 0.00064 | 0.00065 | 0.00077 | 0.00024 |
| MW020 | 0 | 287886 | 2164 | 1 | 0.00059 | 0.0003 | 0.00059 | 0 |
| MW028 | 0 | 347111 | 2715 | 1.8703 | 0.00071 | 0.00055 | 0.00079 | 0.00012 |
| MW019 | 0 | 351087 | 2795 | 3.4859 | 0.00064 | 0.0006 | 0.00073 | 0.00016 |
| MW004 | 0 | 351347 | 2823 | 3.53834 | 0.00064 | 0.00067 | 0.00079 | 0.00029 |
| MW021 | 1 | 348208 | 2558 | 3.91797 | 0.00054 | 0.00054 | 0.00063 | 0.00018 |
| MW017 | 0 | 351587 | 2789 | 3.44872 | 0.00063 | 0.0006 | 0.00071 | 0.00014 |
| MW051 | 0 | 341263 | 2726 | 1.85324 | 0.00065 | 0.00049 | 0.00069 | 0.00006 |
| MW007 | 0 | 346644 | 2761 | 4.02627 | 0.00056 | 0.00067 | 0.00078 | 0.00044 |
| MW056 | 0 | 343500 | 2701 | 2.58893 | 0.00059 | 0.00053 | 0.00068 | 0.00015 |
| MW057 | 1 | 352319 | 2832 | 4.2405 | 0.00067 | 0.00071 | 0.00082 | 0.00031 |
| MW027 | 0 | 349159 | 2776 | 2.66328 | 0.00068 | 0.00058 | 0.00073 | 0.00008 |
| MW049 | 2 | 351927 | 2824 | 4.35672 | 0.00066 | 0.00065 | 0.00074 | 0.00017 |
| MW077 | 3 | 350149 | 2796 | 4.11653 | 0.00065 | 0.00064 | 0.00075 | 0.00021 |
| MW039 | 0 | 351668 | 2827 | 4.26166 | 0.00062 | 0.00072 | 0.00083 | 0.00041 |
| MW001 | 2 | 351453 | 2814 | 4.11169 | 0.00063 | 0.00065 | 0.00077 | 0.00028 |
| MW006 | 0 | 352177 | 2826 | 4.31482 | 0.00065 | 0.00073 | 0.00084 | 0.0004 |
| MW072 | 1 | 351467 | 2810 | 4.20399 | 0.00064 | 0.00068 | 0.00078 | 0.00029 |
| MW023 | 0 | 304597 | 2274 | 1 | 0.00066 | 0.00033 | 0.00066 | 0 |
| MW036 | 2 | 349999 | 2818 | 3.47636 | 0.00065 | 0.00067 | 0.00079 | 0.00027 |
| MW062 | 0 | 350628 | 2808 | 4.16038 | 0.00065 | 0.0007 | 0.00081 | 0.00032 |
| MW045 | 0 | 322174 | 2391 | 1 | 0.00061 | 0.0003 | 0.00061 | 0 |
| MW016 | 0 | 316088 | 1730 | 1 | 0.00047 | 0.00024 | 0.00047 | 0 |
| MW046 | 0 | 352252 | 2801 | 4.31529 | 0.00061 | 0.00064 | 0.00074 | 0.00025 |
| MW061 | 0 | 347015 | 2791 | 2.66839 | 0.00068 | 0.00058 | 0.00074 | 0.00008 |
| MW068 | 0 | 351784 | 2796 | 4.1996 | 0.0006 | 0.00061 | 0.00071 | 0.00021 |
| MW029 | 0 | 346144 | 2724 | 2.59084 | 0.00059 | 0.00057 | 0.00073 | 0.00023 |
| MW014 | 0 | 351056 | 2814 | 4.16209 | 0.00061 | 0.00072 | 0.00082 | 0.00044 |
| MW042 | 0 | 340527 | 2730 | 1.91736 | 0.00065 | 0.00048 | 0.00067 | 0.00003 |
| MW003 | 0 | 349709 | 2755 | 3.37607 | 0.00055 | 0.00052 | 0.00064 | 0.00015 |
| MW031 | 1 | 351197 | 2829 | 4.18191 | 0.00063 | 0.00073 | 0.00084 | 0.00044 |
| MW015 | 0 | 350370 | 2761 | 3.53846 | 0.00064 | 0.00066 | 0.0008 | 0.00028 |
| MW009 | 0 | 337933 | 2665 | 1.79103 | 0.00061 | 0.00051 | 0.00071 | 0.00016 |
| MW011 | 1 | 349651 | 2785 | 3.41658 | 0.00059 | 0.00058 | 0.00071 | 0.00021 |
| MW064 | 1 | 347900 | 2781 | 2.6193 | 0.00065 | 0.00059 | 0.00073 | 0.00015 |
| MW069 | 0 | 344707 | 2728 | 1.85459 | 0.00065 | 0.00048 | 0.00068 | 0.00004 |
| MW035 | 0 | 350584 | 2796 | 3.52808 | 0.00062 | 0.00062 | 0.00074 | 0.00023 |
| MW008 | 0 | 347798 | 2729 | 2.69047 | 0.00067 | 0.00057 | 0.00074 | 0.0001 |
| MW005 | 1 | 352711 | 2815 | 4.46654 | 0.00066 | 0.00073 | 0.00083 | 0.00035 |
| MW074 | 0 | 334912 | 2521 | 2.41836 | 0.00052 | 0.00051 | 0.00065 | 0.00022 |
| MW041 | 0 | 345238 | 2695 | 1.86941 | 0.0006 | 0.00048 | 0.00068 | 0.00012 |
| MWI067 | 0 | 323332 | 2457 | 1 | 0.00059 | 0.00029 | 0.00059 | 0 |
| MWI075 | 0 | 302808 | 2412 | 1 | 0.00067 | 0.00033 | 0.00067 | 0 |
| MWI007 | 0 | 299066 | 2449 | 1 | 0.00071 | 0.00035 | 0.00071 | 0 |
| MWI016 | 0 | 301183 | 2472 | 1 | 0.00066 | 0.00033 | 0.00066 | 0 |
| MWI025 | 0 | 290975 | 2389 | 1 | 0.00061 | 0.0003 | 0.00061 | 0 |
| MWI033 | 0 | 326371 | 2503 | 1 | 0.00053 | 0.00027 | 0.00053 | 0 |
| MWI041 | 0 | 327036 | 2565 | 1 | 0.00064 | 0.00032 | 0.00064 | 0 |
| MWI049 | 0 | 312058 | 1711 | 1 | 0.00054 | 0.00027 | 0.00054 | 0 |
| MWI058 | 0 | 319512 | 2493 | 1 | 0.00069 | 0.00035 | 0.00069 | 0 |
| MWI068 | 0 | 313803 | 2291 | 1 | 0.00056 | 0.00028 | 0.00056 | 0 |
| MWI076 | 0 | 290689 | 2416 | 1 | 0.0007 | 0.00035 | 0.0007 | 0 |
| MW026 | 0 | 326203 | 2458 | 1 | 0.00063 | 0.00031 | 0.00063 | 0 |
| MWI008 | 0 | 326965 | 2524 | 1 | 0.00062 | 0.00031 | 0.00062 | 0 |
| MWI017 | 0 | 312005 | 2473 | 1 | 0.00068 | 0.00034 | 0.00068 | 0 |
| MWI026 | 0 | 308306 | 2506 | 1 | 0.00065 | 0.00032 | 0.00065 | 0 |
| MWI034 | 0 | 260935 | 1880 | 1 | 0.00057 | 0.00029 | 0.00057 | 0 |
| MWI042 | 0 | 315842 | 1641 | 1 | 0.00049 | 0.00024 | 0.00049 | 0 |
| MWI050 | 0 | 308828 | 1656 | 1 | 0.0005 | 0.00025 | 0.0005 | 0 |
| MWI059 | 0 | 321163 | 2002 | 1 | 0.00048 | 0.00024 | 0.00048 | 0 |
| MWI069 | 0 | 316877 | 2533 | 1 | 0.00069 | 0.00034 | 0.00069 | 0 |
| MWI077 | 0 | 310446 | 2052 | 1 | 0.00058 | 0.00029 | 0.00058 | 0 |
| MWI009 | 0 | 266913 | 1640 | 1 | 0.0006 | 0.0003 | 0.0006 | 0 |
| MWI018 | 0 | 324891 | 2485 | 1 | 0.00077 | 0.00038 | 0.00077 | 0 |
| MWI027 | 0 | 318788 | 2460 | 1 | 0.00085 | 0.00043 | 0.00085 | 0 |
| MWI035 | 0 | 254439 | 1811 | 1 | 0.00061 | 0.00031 | 0.00061 | 0 |
| MWI043 | 0 | 305390 | 1742 | 1 | 0.00044 | 0.00022 | 0.00044 | 0 |
| MWI051 | 0 | 325761 | 1929 | 1 | 0.00049 | 0.00025 | 0.00049 | 0 |
| MWI060 | 0 | 329739 | 2537 | 1 | 0.00063 | 0.00031 | 0.00063 | 0 |
| MWI070 | 0 | 337035 | 2532 | 1 | 0.0006 | 0.0003 | 0.0006 | 0 |
| MWI078 | 0 | 329827 | 2547 | 1 | 0.00072 | 0.00036 | 0.00072 | 0 |
| MWI010 | 0 | 331096 | 2516 | 1 | 0.00062 | 0.00031 | 0.00062 | 0 |
| MWI019 | 0 | 293410 | 2381 | 1 | 0.00061 | 0.00031 | 0.00061 | 0 |
| MWI028 | 0 | 279911 | 1956 | 1 | 0.00059 | 0.00029 | 0.00059 | 0 |
| MWI036 | 0 | 318090 | 2471 | 1 | 0.00069 | 0.00035 | 0.00069 | 0 |
| MWI044 | 0 | 335574 | 2549 | 1 | 0.00063 | 0.00032 | 0.00063 | 0 |
| MWI052 | 0 | 284824 | 2379 | 1 | 0.0006 | 0.0003 | 0.0006 | 0 |
| MWI061 | 0 | 334310 | 2581 | 1 | 0.00093 | 0.00047 | 0.00093 | 0 |
| MWI071 | 0 | 317981 | 2486 | 1 | 0.00063 | 0.00032 | 0.00063 | 0 |
| MWI079 | 0 | 311865 | 2548 | 1 | 0.00068 | 0.00034 | 0.00068 | 0 |
| MWI001 | 0 | 310619 | 2098 | 1 | 0.00047 | 0.00024 | 0.00047 | 0 |
| MWI011 | 0 | 320478 | 2500 | 1 | 0.00059 | 0.00029 | 0.00059 | 0 |
| MWI020 | 0 | 249171 | 1868 | 1 | 0.00057 | 0.00029 | 0.00057 | 0 |
| MWI029 | 0 | 330390 | 2496 | 1 | 0.00061 | 0.00031 | 0.00061 | 0 |
| MWI045 | 0 | 322227 | 2148 | 1 | 0.00075 | 0.00038 | 0.00075 | 0 |
| MWI053 | 0 | 291388 | 1355 | 1 | 0.00054 | 0.00027 | 0.00054 | 0 |
| MWI062 | 0 | 322349 | 2474 | 1 | 0.00073 | 0.00036 | 0.00073 | 0 |
| MWI072 | 0 | 299043 | 2420 | 1 | 0.00073 | 0.00036 | 0.00073 | 0 |
| MWI080 | 0 | 289251 | 1886 | 1 | 0.00047 | 0.00023 | 0.00047 | 0 |
| MWI003 | 0 | 317790 | 2430 | 1 | 0.00065 | 0.00032 | 0.00065 | 0 |
| MWI014 | 0 | 320763 | 2491 | 1 | 0.00066 | 0.00033 | 0.00066 | 0 |
| MWI022 | 0 | 235120 | 1934 | 1 | 0.00069 | 0.00034 | 0.00069 | 0 |
| MWI030 | 0 | 326673 | 2360 | 1 | 0.0006 | 0.0003 | 0.0006 | 0 |
| MWI038 | 0 | 319822 | 2256 | 1 | 0.00061 | 0.0003 | 0.00061 | 0 |
| MWI046 | 0 | 309869 | 2478 | 1 | 0.00077 | 0.00039 | 0.00077 | 0 |
| MWI054 | 0 | 280893 | 1412 | 1 | 0.0005 | 0.00025 | 0.0005 | 0 |
| MWI063 | 0 | 329579 | 2509 | 1 | 0.00064 | 0.00032 | 0.00064 | 0 |
| MWI073 | 0 | 306285 | 2534 | 1 | 0.00071 | 0.00036 | 0.00071 | 0 |
| UTSC | 0 | 297296 | 2305 | 1 | 0.00061 | 0.0003 | 0.00061 | 0 |
| MWI004 | 0 | 310993 | 1632 | 1 | 0.00048 | 0.00024 | 0.00048 | 0 |
| MWI015 | 0 | 286293 | 2349 | 1 | 0.00065 | 0.00032 | 0.00065 | 0 |
| MWI023 | 0 | 333518 | 2516 | 1 | 0.00063 | 0.00032 | 0.00063 | 0 |
| MWI031 | 0 | 313671 | 1913 | 1 | 0.00048 | 0.00024 | 0.00048 | 0 |
| MWI039 | 0 | 309865 | 2510 | 1 | 0.00065 | 0.00032 | 0.00065 | 0 |
| MWI047 | 0 | 306935 | 2527 | 1 | 0.00062 | 0.00031 | 0.00062 | 0 |
| MWI055 | 0 | 249641 | 1819 | 1 | 0.00055 | 0.00027 | 0.00055 | 0 |
| MWI066 | 0 | 241924 | 1496 | 1 | 0.00059 | 0.00029 | 0.00059 | 0 |
| MWI074 | 0 | 294488 | 1851 | 1 | 0.00052 | 0.00026 | 0.00052 | 0 |
| MWI005 | 0 | 270506 | 2420 | 1 | 0.00071 | 0.00035 | 0.00071 | 0 |
| MWI024 | 0 | 261156 | 2361 | 1 | 0.00083 | 0.00042 | 0.00083 | 0 |
| MWI032 | 0 | 246473 | 2210 | 1 | 0.00073 | 0.00037 | 0.00073 | 0 |
| MWI040 | 0 | 277604 | 2409 | 1 | 0.00075 | 0.00038 | 0.00075 | 0 |
| MWI048 | 0 | 279867 | 2436 | 1 | 0.00076 | 0.00038 | 0.00076 | 0 |
| MWI056 | 0 | 181209 | 1625 | 1 | 0.00084 | 0.00042 | 0.00084 | 0 |

Table S4

Stacks summary statistics for variant positions within dataset #2. The following parameters are shown for each sampling site: private alleles, mean individuals per locus, mean observed (Ho) and expected (He) heterozygosity, mean nucleotide diversity (π), and mean inbreeding coefficient (FIS).

| Sampling Site | Private | Individuals | Ho | He | π | FIS |
| --- | --- | --- | --- | --- | --- | --- |
| MW067 | 0 | 3.72285 | 0.20993 | 0.18805 | 0.21793 | 0.01522 |
| MW079 | 0 | 4.62449 | 0.21089 | 0.2188 | 0.24575 | 0.07584 |
| MW040 | 0 | 3.64985 | 0.22434 | 0.20766 | 0.24244 | 0.03462 |
| MW047 | 0 | 4.64264 | 0.21782 | 0.2067 | 0.23231 | 0.03455 |
| MW002 | 0 | 4.67798 | 0.21862 | 0.22078 | 0.24748 | 0.06449 |
| MW012 | 0 | 4.67868 | 0.22075 | 0.19603 | 0.22056 | 0.00103 |
| MW013 | 0 | 2.78328 | 0.22308 | 0.17984 | 0.22081 | -0.00335 |
| MW018 | 0 | 3.67593 | 0.24854 | 0.22188 | 0.25838 | 0.01869 |
| MW030 | 0 | 3.69928 | 0.22262 | 0.20007 | 0.23263 | 0.02122 |
| MW020 | 0 | 1 | 0.20829 | 0.10414 | 0.20829 | 0 |
| MW028 | 0 | 1.87539 | 0.22222 | 0.16667 | 0.2288 | 0.00987 |
| MW019 | 0 | 3.77984 | 0.20765 | 0.18288 | 0.21176 | 0.00818 |
| MW004 | 0 | 3.7716 | 0.21519 | 0.20723 | 0.24014 | 0.05072 |
| MW021 | 0 | 4.19251 | 0.20628 | 0.19718 | 0.22625 | 0.03983 |
| MW017 | 0 | 3.7034 | 0.22134 | 0.19571 | 0.22813 | 0.0112 |
| MW051 | 0 | 1.8875 | 0.22083 | 0.15807 | 0.21806 | -0.00417 |
| MW007 | 0 | 4.4243 | 0.19252 | 0.21159 | 0.23986 | 0.09875 |
| MW056 | 0 | 2.68672 | 0.20695 | 0.18184 | 0.22521 | 0.03256 |
| MW057 | 0 | 4.62037 | 0.21867 | 0.21539 | 0.24234 | 0.05296 |
| MW027 | 0 | 2.81011 | 0.23099 | 0.18506 | 0.22666 | -0.01009 |
| MW049 | 0 | 4.68519 | 0.21864 | 0.19634 | 0.22167 | 0.0086 |
| MW077 | 0 | 4.47271 | 0.20374 | 0.18941 | 0.21475 | 0.02771 |
| MW039 | 0 | 4.66152 | 0.22286 | 0.22486 | 0.25301 | 0.06322 |
| MW001 | 0 | 4.42798 | 0.20298 | 0.19167 | 0.21717 | 0.0319 |
| MW006 | 0 | 4.65329 | 0.2113 | 0.2134 | 0.23939 | 0.0607 |
| MW072 | 0 | 4.64367 | 0.22089 | 0.21397 | 0.24062 | 0.04308 |
| MW023 | 0 | 1 | 0.2183 | 0.10915 | 0.2183 | 0 |
| MW036 | 0 | 3.76132 | 0.21982 | 0.2032 | 0.2357 | 0.03374 |
| MW062 | 0 | 4.5463 | 0.22013 | 0.20741 | 0.23368 | 0.02939 |
| MW045 | 0 | 1 | 0.22566 | 0.11283 | 0.22566 | 0 |
| MW016 | 0 | 1 | 0.20324 | 0.10162 | 0.20324 | 0 |
| MW046 | 0 | 4.52268 | 0.20931 | 0.20105 | 0.22644 | 0.03721 |
| MW061 | 0 | 2.81649 | 0.23265 | 0.18196 | 0.22454 | -0.01458 |
| MW068 | 0 | 4.57819 | 0.19952 | 0.18482 | 0.20832 | 0.02083 |
| MW029 | 0 | 2.71044 | 0.20924 | 0.18577 | 0.22923 | 0.03338 |
| MW014 | 0 | 4.62925 | 0.22072 | 0.22669 | 0.25489 | 0.07368 |
| MW042 | 0 | 1.89572 | 0.19864 | 0.14038 | 0.19517 | -0.00521 |
| MW003 | 0 | 3.55005 | 0.20253 | 0.17265 | 0.2036 | -0.00038 |
| MW031 | 0 | 4.63374 | 0.20396 | 0.21765 | 0.24426 | 0.09156 |
| MW015 | 0 | 3.59465 | 0.22308 | 0.2127 | 0.24951 | 0.05102 |
| MW009 | 0 | 1.87006 | 0.22193 | 0.1784 | 0.2455 | 0.03534 |
| MW011 | 0 | 3.6241 | 0.201 | 0.18636 | 0.21764 | 0.03091 |
| MW064 | 0 | 2.82972 | 0.22205 | 0.19965 | 0.24283 | 0.03615 |
| MW069 | 0 | 1.91675 | 0.20447 | 0.14347 | 0.1958 | -0.01301 |
| MW035 | 0 | 3.6931 | 0.22108 | 0.19838 | 0.23014 | 0.01941 |
| MW008 | 0 | 2.67389 | 0.23048 | 0.19192 | 0.23722 | 0.01009 |
| MW005 | 0 | 4.65123 | 0.21824 | 0.21863 | 0.24565 | 0.06035 |
| MW074 | 0 | 2.63285 | 0.17538 | 0.16986 | 0.21032 | 0.06029 |
| MW041 | 0 | 1.89256 | 0.21384 | 0.17149 | 0.23623 | 0.03357 |
| MWI067 | 0 | 1 | 0.19826 | 0.09913 | 0.19826 | 0 |
| MWI075 | 0 | 1 | 0.22472 | 0.11236 | 0.22472 | 0 |
| MWI007 | 0 | 1 | 0.21922 | 0.10961 | 0.21922 | 0 |
| MWI016 | 0 | 1 | 0.21007 | 0.10503 | 0.21007 | 0 |
| MWI025 | 0 | 1 | 0.21786 | 0.10893 | 0.21786 | 0 |
| MWI033 | 0 | 1 | 0.20824 | 0.10412 | 0.20824 | 0 |
| MWI041 | 0 | 1 | 0.20751 | 0.10375 | 0.20751 | 0 |
| MWI049 | 0 | 1 | 0.22882 | 0.11441 | 0.22882 | 0 |
| MWI058 | 0 | 1 | 0.22064 | 0.11032 | 0.22064 | 0 |
| MWI068 | 0 | 1 | 0.22772 | 0.11386 | 0.22772 | 0 |
| MWI076 | 0 | 1 | 0.22538 | 0.11269 | 0.22538 | 0 |
| MW026 | 0 | 1 | 0.22332 | 0.11166 | 0.22332 | 0 |
| MWI008 | 0 | 1 | 0.18212 | 0.09106 | 0.18212 | 0 |
| MWI017 | 0 | 1 | 0.22433 | 0.11217 | 0.22433 | 0 |
| MWI026 | 0 | 1 | 0.21729 | 0.10865 | 0.21729 | 0 |
| MWI034 | 0 | 1 | 0.18721 | 0.0936 | 0.18721 | 0 |
| MWI042 | 0 | 1 | 0.21867 | 0.10934 | 0.21867 | 0 |
| MWI050 | 0 | 1 | 0.22292 | 0.11146 | 0.22292 | 0 |
| MWI059 | 0 | 1 | 0.20455 | 0.10227 | 0.20455 | 0 |
| MWI069 | 0 | 1 | 0.22595 | 0.11297 | 0.22595 | 0 |
| MWI077 | 0 | 1 | 0.2007 | 0.10035 | 0.2007 | 0 |
| MWI009 | 0 | 1 | 0.2046 | 0.1023 | 0.2046 | 0 |
| MWI018 | 0 | 1 | 0.23203 | 0.11601 | 0.23203 | 0 |
| MWI027 | 0 | 1 | 0.32302 | 0.16151 | 0.32302 | 0 |
| MWI035 | 0 | 1 | 0.22439 | 0.1122 | 0.22439 | 0 |
| MWI043 | 0 | 1 | 0.18642 | 0.09321 | 0.18642 | 0 |
| MWI051 | 0 | 1 | 0.22581 | 0.1129 | 0.22581 | 0 |
| MWI060 | 0 | 1 | 0.20087 | 0.10043 | 0.20087 | 0 |
| MWI070 | 0 | 1 | 0.20883 | 0.10441 | 0.20883 | 0 |
| MWI078 | 0 | 1 | 0.24269 | 0.12134 | 0.24269 | 0 |
| MWI010 | 0 | 1 | 0.21328 | 0.10664 | 0.21328 | 0 |
| MWI019 | 0 | 1 | 0.22039 | 0.1102 | 0.22039 | 0 |
| MWI028 | 0 | 1 | 0.18638 | 0.09319 | 0.18638 | 0 |
| MWI036 | 0 | 1 | 0.22732 | 0.11366 | 0.22732 | 0 |
| MWI044 | 0 | 1 | 0.18281 | 0.0914 | 0.18281 | 0 |
| MWI052 | 0 | 1 | 0.2064 | 0.1032 | 0.2064 | 0 |
| MWI061 | 0 | 1 | 0.35187 | 0.17594 | 0.35187 | 0 |
| MWI071 | 0 | 1 | 0.19417 | 0.09709 | 0.19417 | 0 |
| MWI079 | 0 | 1 | 0.22668 | 0.11334 | 0.22668 | 0 |
| MWI001 | 0 | 1 | 0.1984 | 0.0992 | 0.1984 | 0 |
| MWI011 | 0 | 1 | 0.19116 | 0.09558 | 0.19116 | 0 |
| MWI020 | 0 | 1 | 0.1831 | 0.09155 | 0.1831 | 0 |
| MWI029 | 0 | 1 | 0.21701 | 0.10851 | 0.21701 | 0 |
| MWI045 | 0 | 1 | 0.30966 | 0.15483 | 0.30966 | 0 |
| MWI053 | 0 | 1 | 0.25884 | 0.12942 | 0.25884 | 0 |
| MWI062 | 0 | 1 | 0.25108 | 0.12554 | 0.25108 | 0 |
| MWI072 | 0 | 1 | 0.21643 | 0.10821 | 0.21643 | 0 |
| MWI080 | 0 | 1 | 0.17949 | 0.08974 | 0.17949 | 0 |
| MWI003 | 0 | 1 | 0.20174 | 0.10087 | 0.20174 | 0 |
| MWI014 | 0 | 1 | 0.2311 | 0.11555 | 0.2311 | 0 |
| MWI022 | 0 | 1 | 0.20561 | 0.1028 | 0.20561 | 0 |
| MWI030 | 0 | 1 | 0.22429 | 0.11214 | 0.22429 | 0 |
| MWI038 | 0 | 1 | 0.20907 | 0.10454 | 0.20907 | 0 |
| MWI046 | 0 | 1 | 0.23286 | 0.11643 | 0.23286 | 0 |
| MWI054 | 0 | 1 | 0.24205 | 0.12102 | 0.24205 | 0 |
| MWI063 | 0 | 1 | 0.20066 | 0.10033 | 0.20066 | 0 |
| MWI073 | 0 | 1 | 0.22052 | 0.11026 | 0.22052 | 0 |
| UTSC | 0 | 1 | 0.20705 | 0.10352 | 0.20705 | 0 |
| MWI004 | 0 | 1 | 0.22593 | 0.11297 | 0.22593 | 0 |
| MWI015 | 0 | 1 | 0.22863 | 0.11432 | 0.22863 | 0 |
| MWI023 | 0 | 1 | 0.20455 | 0.10227 | 0.20455 | 0 |
| MWI031 | 0 | 1 | 0.20329 | 0.10165 | 0.20329 | 0 |
| MWI039 | 0 | 1 | 0.20175 | 0.10088 | 0.20175 | 0 |
| MWI047 | 0 | 1 | 0.20915 | 0.10458 | 0.20915 | 0 |
| MWI055 | 0 | 1 | 0.20869 | 0.10434 | 0.20869 | 0 |
| MWI066 | 0 | 1 | 0.2122 | 0.1061 | 0.2122 | 0 |
| MWI074 | 0 | 1 | 0.18992 | 0.09496 | 0.18992 | 0 |
| MWI005 | 0 | 1 | 0.20611 | 0.10305 | 0.20611 | 0 |
| MWI024 | 0 | 1 | 0.22418 | 0.11209 | 0.22418 | 0 |
| MWI032 | 0 | 1 | 0.20971 | 0.10486 | 0.20971 | 0 |
| MWI040 | 0 | 1 | 0.24615 | 0.12308 | 0.24615 | 0 |
| MWI048 | 0 | 1 | 0.21507 | 0.10753 | 0.21507 | 0 |
| MWI056 | 0 | 1 | 0.22468 | 0.11234 | 0.22468 | 0 |

Table S5

Stacks summary statistics for all positions (variant and fixed) within dataset #2. The following parameters are shown for each sampling site: private alleles, total sites, variant sites, mean individuals per locus, mean observed (Ho) and expected (He) heterozygosity, mean nucleotide diversity (π), and mean inbreeding coefficient (FIS).

| Sampling Site | Private | Sites | Variant sites | Individuals | Ho | He | π | FIS |
| --- | --- | --- | --- | --- | --- | --- | --- | --- |
| MW067 | 0 | 259423 | 967 | 3.73036 | 0.00078 | 0.0007 | 0.00081 | 0.00006 |
| MW079 | 0 | 259484 | 972 | 4.81874 | 0.00079 | 0.00082 | 0.00092 | 0.00028 |
| MW040 | 0 | 259376 | 971 | 3.69825 | 0.00084 | 0.00078 | 0.00091 | 0.00013 |
| MW047 | 0 | 259483 | 971 | 4.75908 | 0.00082 | 0.00077 | 0.00087 | 0.00013 |
| MW002 | 0 | 259484 | 972 | 4.80321 | 0.00082 | 0.00083 | 0.00093 | 0.00024 |
| MW012 | 0 | 259483 | 971 | 4.76723 | 0.00083 | 0.00073 | 0.00083 | 0 |
| MW013 | 0 | 259481 | 969 | 2.83298 | 0.00083 | 0.00067 | 0.00082 | -0.00001 |
| MW018 | 0 | 259484 | 972 | 3.80893 | 0.00093 | 0.00083 | 0.00097 | 0.00007 |
| MW030 | 0 | 259389 | 971 | 3.75802 | 0.00083 | 0.00075 | 0.00087 | 0.00008 |
| MW020 | 0 | 241627 | 893 | 1 | 0.00077 | 0.00038 | 0.00077 | 0 |
| MW028 | 0 | 259000 | 963 | 1.95536 | 0.00083 | 0.00062 | 0.00085 | 0.00004 |
| MW019 | 0 | 259189 | 972 | 3.81346 | 0.00078 | 0.00069 | 0.00079 | 0.00003 |
| MW004 | 0 | 259394 | 972 | 3.82323 | 0.00081 | 0.00078 | 0.0009 | 0.00019 |
| MW021 | 0 | 259473 | 961 | 4.39168 | 0.00076 | 0.00073 | 0.00084 | 0.00015 |
| MW017 | 0 | 259483 | 971 | 3.77315 | 0.00083 | 0.00073 | 0.00085 | 0.00004 |
| MW051 | 0 | 258714 | 960 | 1.94796 | 0.00082 | 0.00059 | 0.00081 | -0.00002 |
| MW007 | 0 | 259055 | 971 | 4.54662 | 0.00072 | 0.00079 | 0.0009 | 0.00037 |
| MW056 | 0 | 259003 | 964 | 2.80689 | 0.00077 | 0.00068 | 0.00084 | 0.00012 |
| MW057 | 0 | 259484 | 972 | 4.6343 | 0.00082 | 0.00081 | 0.00091 | 0.0002 |
| MW027 | 0 | 259481 | 969 | 2.85742 | 0.00086 | 0.00069 | 0.00085 | -0.00004 |
| MW049 | 0 | 259484 | 972 | 4.75257 | 0.00082 | 0.00074 | 0.00083 | 0.00003 |
| MW077 | 0 | 259121 | 971 | 4.55172 | 0.00076 | 0.00071 | 0.0008 | 0.0001 |
| MW039 | 0 | 259484 | 972 | 4.68723 | 0.00083 | 0.00084 | 0.00095 | 0.00024 |
| MW001 | 0 | 259484 | 972 | 4.57368 | 0.00076 | 0.00072 | 0.00081 | 0.00012 |
| MW006 | 0 | 259484 | 972 | 4.71108 | 0.00079 | 0.0008 | 0.0009 | 0.00023 |
| MW072 | 0 | 259483 | 971 | 4.66185 | 0.00083 | 0.0008 | 0.0009 | 0.00016 |
| MW023 | 0 | 247341 | 907 | 1 | 0.0008 | 0.0004 | 0.0008 | 0 |
| MW036 | 0 | 259422 | 972 | 3.7957 | 0.00082 | 0.00076 | 0.00088 | 0.00013 |
| MW062 | 0 | 259484 | 972 | 4.64876 | 0.00082 | 0.00078 | 0.00088 | 0.00011 |
| MW045 | 0 | 252545 | 904 | 1 | 0.00081 | 0.0004 | 0.00081 | 0 |
| MW016 | 0 | 249819 | 802 | 1 | 0.00065 | 0.00033 | 0.00065 | 0 |
| MW046 | 0 | 259482 | 970 | 4.75704 | 0.00078 | 0.00075 | 0.00085 | 0.00014 |
| MW061 | 0 | 259174 | 970 | 2.86131 | 0.00087 | 0.00068 | 0.00084 | -0.00005 |
| MW068 | 0 | 259484 | 972 | 4.6508 | 0.00075 | 0.00069 | 0.00078 | 0.00008 |
| MW029 | 0 | 259125 | 967 | 2.8269 | 0.00078 | 0.00069 | 0.00086 | 0.00012 |
| MW014 | 0 | 259483 | 971 | 4.63067 | 0.00083 | 0.00085 | 0.00095 | 0.00028 |
| MW042 | 0 | 258243 | 959 | 1.96631 | 0.00074 | 0.00052 | 0.00072 | -0.00002 |
| MW003 | 0 | 259421 | 969 | 3.73137 | 0.00076 | 0.00064 | 0.00076 | 0 |
| MW031 | 0 | 259484 | 972 | 4.64921 | 0.00076 | 0.00082 | 0.00091 | 0.00034 |
| MW015 | 0 | 259362 | 972 | 3.8136 | 0.00084 | 0.0008 | 0.00094 | 0.00019 |
| MW009 | 0 | 257554 | 962 | 1.90954 | 0.00083 | 0.00067 | 0.00092 | 0.00013 |
| MW011 | 0 | 259383 | 971 | 3.72738 | 0.00075 | 0.0007 | 0.00081 | 0.00012 |
| MW064 | 0 | 259025 | 969 | 2.83766 | 0.00083 | 0.00075 | 0.00091 | 0.00014 |
| MW069 | 0 | 258756 | 961 | 1.94429 | 0.00076 | 0.00053 | 0.00073 | -0.00005 |
| MW035 | 0 | 259411 | 971 | 3.83 | 0.00083 | 0.00074 | 0.00086 | 0.00007 |
| MW008 | 0 | 259106 | 969 | 2.88843 | 0.00086 | 0.00072 | 0.00089 | 0.00004 |
| MW005 | 0 | 259484 | 972 | 4.82523 | 0.00082 | 0.00082 | 0.00092 | 0.00023 |
| MW074 | 0 | 257103 | 956 | 2.64322 | 0.00065 | 0.00063 | 0.00078 | 0.00022 |
| MW041 | 0 | 258667 | 968 | 1.95887 | 0.0008 | 0.00064 | 0.00088 | 0.00013 |
| MWI067 | 0 | 253638 | 918 | 1 | 0.00072 | 0.00036 | 0.00072 | 0 |
| MWI075 | 0 | 246197 | 890 | 1 | 0.00081 | 0.00041 | 0.00081 | 0 |
| MWI007 | 0 | 243818 | 926 | 1 | 0.00083 | 0.00042 | 0.00083 | 0 |
| MWI016 | 0 | 245570 | 914 | 1 | 0.00078 | 0.00039 | 0.00078 | 0 |
| MWI025 | 0 | 241905 | 918 | 1 | 0.00083 | 0.00041 | 0.00083 | 0 |
| MWI033 | 0 | 254918 | 922 | 1 | 0.00075 | 0.00038 | 0.00075 | 0 |
| MWI041 | 0 | 253154 | 906 | 1 | 0.00074 | 0.00037 | 0.00074 | 0 |
| MWI049 | 0 | 248126 | 791 | 1 | 0.00073 | 0.00036 | 0.00073 | 0 |
| MWI058 | 0 | 252188 | 911 | 1 | 0.0008 | 0.0004 | 0.0008 | 0 |
| MWI068 | 0 | 251353 | 909 | 1 | 0.00082 | 0.00041 | 0.00082 | 0 |
| MWI076 | 0 | 242202 | 914 | 1 | 0.00085 | 0.00043 | 0.00085 | 0 |
| MW026 | 0 | 253490 | 909 | 1 | 0.0008 | 0.0004 | 0.0008 | 0 |
| MWI008 | 0 | 254717 | 906 | 1 | 0.00065 | 0.00032 | 0.00065 | 0 |
| MWI017 | 0 | 248825 | 896 | 1 | 0.00081 | 0.0004 | 0.00081 | 0 |
| MWI026 | 0 | 247969 | 902 | 1 | 0.00079 | 0.0004 | 0.00079 | 0 |
| MWI034 | 0 | 220477 | 860 | 1 | 0.00073 | 0.00037 | 0.00073 | 0 |
| MWI042 | 0 | 248911 | 782 | 1 | 0.00069 | 0.00034 | 0.00069 | 0 |
| MWI050 | 0 | 247955 | 794 | 1 | 0.00071 | 0.00036 | 0.00071 | 0 |
| MWI059 | 0 | 252395 | 880 | 1 | 0.00071 | 0.00036 | 0.00071 | 0 |
| MWI069 | 0 | 252600 | 925 | 1 | 0.00083 | 0.00041 | 0.00083 | 0 |
| MWI077 | 0 | 249750 | 862 | 1 | 0.00069 | 0.00035 | 0.00069 | 0 |
| MWI009 | 0 | 222516 | 782 | 1 | 0.00072 | 0.00036 | 0.00072 | 0 |
| MWI018 | 0 | 254360 | 918 | 1 | 0.00084 | 0.00042 | 0.00084 | 0 |
| MWI027 | 0 | 253829 | 873 | 1 | 0.00111 | 0.00056 | 0.00111 | 0 |
| MWI035 | 0 | 217937 | 820 | 1 | 0.00084 | 0.00042 | 0.00084 | 0 |
| MWI043 | 0 | 245754 | 810 | 1 | 0.00061 | 0.00031 | 0.00061 | 0 |
| MWI051 | 0 | 252715 | 837 | 1 | 0.00075 | 0.00037 | 0.00075 | 0 |
| MWI060 | 0 | 255850 | 921 | 1 | 0.00072 | 0.00036 | 0.00072 | 0 |
| MWI070 | 0 | 257250 | 929 | 1 | 0.00075 | 0.00038 | 0.00075 | 0 |
| MWI078 | 0 | 254538 | 923 | 1 | 0.00088 | 0.00044 | 0.00088 | 0 |
| MWI010 | 0 | 255277 | 919 | 1 | 0.00077 | 0.00038 | 0.00077 | 0 |
| MWI019 | 0 | 243171 | 912 | 1 | 0.00083 | 0.00041 | 0.00083 | 0 |
| MWI028 | 0 | 234944 | 837 | 1 | 0.00066 | 0.00033 | 0.00066 | 0 |
| MWI036 | 0 | 251238 | 915 | 1 | 0.00083 | 0.00041 | 0.00083 | 0 |
| MWI044 | 0 | 256049 | 919 | 1 | 0.00066 | 0.00033 | 0.00066 | 0 |
| MWI052 | 0 | 239283 | 906 | 1 | 0.00078 | 0.00039 | 0.00078 | 0 |
| MWI061 | 0 | 256133 | 881 | 1 | 0.00121 | 0.00061 | 0.00121 | 0 |
| MWI071 | 0 | 252077 | 927 | 1 | 0.00071 | 0.00036 | 0.00071 | 0 |
| MWI079 | 0 | 247707 | 922 | 1 | 0.00084 | 0.00042 | 0.00084 | 0 |
| MWI001 | 0 | 249732 | 877 | 1 | 0.0007 | 0.00035 | 0.0007 | 0 |
| MWI011 | 0 | 253602 | 905 | 1 | 0.00068 | 0.00034 | 0.00068 | 0 |
| MWI020 | 0 | 218659 | 852 | 1 | 0.00071 | 0.00036 | 0.00071 | 0 |
| MWI029 | 0 | 255482 | 917 | 1 | 0.00078 | 0.00039 | 0.00078 | 0 |
| MWI045 | 0 | 253971 | 859 | 1 | 0.00105 | 0.00052 | 0.00105 | 0 |
| MWI053 | 0 | 240658 | 707 | 1 | 0.00076 | 0.00038 | 0.00076 | 0 |
| MWI062 | 0 | 252671 | 924 | 1 | 0.00092 | 0.00046 | 0.00092 | 0 |
| MWI072 | 0 | 246061 | 901 | 1 | 0.00079 | 0.0004 | 0.00079 | 0 |
| MWI080 | 0 | 239343 | 819 | 1 | 0.00061 | 0.00031 | 0.00061 | 0 |
| MWI003 | 0 | 251300 | 917 | 1 | 0.00074 | 0.00037 | 0.00074 | 0 |
| MWI014 | 0 | 252382 | 926 | 1 | 0.00085 | 0.00042 | 0.00085 | 0 |
| MWI022 | 0 | 205855 | 856 | 1 | 0.00085 | 0.00043 | 0.00085 | 0 |
| MWI030 | 0 | 253165 | 914 | 1 | 0.00081 | 0.0004 | 0.00081 | 0 |
| MWI038 | 0 | 252153 | 904 | 1 | 0.00075 | 0.00037 | 0.00075 | 0 |
| MWI046 | 0 | 248697 | 919 | 1 | 0.00086 | 0.00043 | 0.00086 | 0 |
| MWI054 | 0 | 234183 | 723 | 1 | 0.00075 | 0.00037 | 0.00075 | 0 |
| MWI063 | 0 | 254027 | 912 | 1 | 0.00072 | 0.00036 | 0.00072 | 0 |
| MWI073 | 0 | 246562 | 916 | 1 | 0.00082 | 0.00041 | 0.00082 | 0 |
| UTSC | 0 | 245059 | 908 | 1 | 0.00077 | 0.00038 | 0.00077 | 0 |
| MWI004 | 0 | 245694 | 779 | 1 | 0.00072 | 0.00036 | 0.00072 | 0 |
| MWI015 | 0 | 238322 | 901 | 1 | 0.00086 | 0.00043 | 0.00086 | 0 |
| MWI023 | 0 | 255327 | 924 | 1 | 0.00074 | 0.00037 | 0.00074 | 0 |
| MWI031 | 0 | 250331 | 851 | 1 | 0.00069 | 0.00035 | 0.00069 | 0 |
| MWI039 | 0 | 249414 | 912 | 1 | 0.00074 | 0.00037 | 0.00074 | 0 |
| MWI047 | 0 | 247305 | 918 | 1 | 0.00078 | 0.00039 | 0.00078 | 0 |
| MWI055 | 0 | 216908 | 829 | 1 | 0.0008 | 0.0004 | 0.0008 | 0 |
| MWI066 | 0 | 210029 | 754 | 1 | 0.00076 | 0.00038 | 0.00076 | 0 |
| MWI074 | 0 | 243917 | 853 | 1 | 0.00066 | 0.00033 | 0.00066 | 0 |
| MWI005 | 0 | 231742 | 917 | 1 | 0.00082 | 0.00041 | 0.00082 | 0 |
| MWI024 | 0 | 226116 | 910 | 1 | 0.0009 | 0.00045 | 0.0009 | 0 |
| MWI032 | 0 | 215111 | 906 | 1 | 0.00088 | 0.00044 | 0.00088 | 0 |
| MWI040 | 0 | 230643 | 910 | 1 | 0.00097 | 0.00049 | 0.00097 | 0 |
| MWI048 | 0 | 233709 | 916 | 1 | 0.00084 | 0.00042 | 0.00084 | 0 |
| MWI056 | 0 | 166199 | 770 | 1 | 0.00104 | 0.00052 | 0.00104 | 0 |

Table S6

Analysis of molecular variance (AMOVA) showing genetic variation sourced from three hierarchical levels when urbanization was classified based on Distance from the City Center. Also shown for each hierarchical level are degrees of freedom, sums of squares, mean squares, variances (sigma), percent variation, and p-values from permutation tests.

| Level | df | SS | MS | Sigma | Variation | p |
| --- | --- | --- | --- | --- | --- | --- |
| Among urban/rural habitats | 1 | 94.068 | 94.068 | 0.022 | 0.032% | 0.375 |
| Among sampling sites within urban/rural habitats | 120 | 9,625.471 | 80.212 | 10.028 | 14.448% | 0.001 |
| Within sampling sites | 134 | 7,954.182 | 59.360 | 59.360 | 85.521% | 0.001 |
| Total | 255 | 17,673.721 | 69.309 | 69.410 | 100% |  |

Table S7

Genetic diversity, FIS, and Tajima’s D calculated for urban and rural habitats when urbanization was classified based on Urbanization Score. Genetic diversity is represented by Watterson’s theta (θW) and nucleotide diversity (π). Tajima’s D was calculated by averaging values from 100 kbp windows, and p-values were calculated with a single-sample t-test testing for deviations from zero.

| Habitat | θW | π | FIS | Tajima’s D | p |
| --- | --- | --- | --- | --- | --- |
| Urban | 0.00146 | 0.00091 | 0.19778 | -0.367 | <0.001 |
| Rural | 0.00151 | 0.00090 | 0.17659 | -0.375 | <0.001 |

Table S8

Results of two-sample t-tests testing for significant differences in Tajima’s D between urban and rural habitats. Shown are the metrics used to classify urbanization, t values, degrees of freedom, and p-values.

| Urbanization | t | df | p |
| --- | --- | --- | --- |
| Distance to City Center | 0.171 | 1,716.327 | 0.864 |
| Urbanization Score | 0.250 | 1,720.650 | 0.802 |

Table S9

Covariance contributed to the spatial conStruct models under K = 1–5 (the number of hypothesized genetic populations).

| K = 1 | K = 2 | K = 3 | K = 4 | K = 5 |
| --- | --- | --- | --- | --- |
| 1 | 0.05 | 0.084 | 0.300 | 0.219 |
| 0 | 0.95 | 0.590 | 0.451 | 0.195 |
| 0 | 0.00 | 0.326 | 0.161 | 0.270 |
| 0 | 0.00 | 0.000 | 0.088 | 0.211 |
| 0 | 0.00 | 0.000 | 0.000 | 0.105 |

Table S10

Cross-validation results for data simulated under K = 1–5 (the number of hypothesized genetic populations), comparing the spatial and nonspatial conStruct models. Higher predictive accuracy values indicate better model performance. Also shown for each model is standard error (SE).

| Model | K | Predictive Accuracy | SE |
| --- | --- | --- | --- |
| Non-spatial | 1 | -55.653 | 4.069 |
| 2 | -48.579 | 4.454 |
| 3 | -76.761 | 5.601 |
| 4 | -81.579 | 6.574 |
| 5 | -76.560 | 6.781 |
| Spatial | 1 | 0.000 | 0.000 |
| 2 | 0.000 | 0.000 |
| 3 | 0.000 | 0.000 |
| 4 | 0.000 | 0.000 |
| 5 | 0.000 | 0.000 |

Table S11

Average pairwise Hudson’s FST between sampling sites within and between each habitat when urbanization was classified based on Urbanization Score.

| Sampling site pair | Mean pairwise Hudson’s FST |
| --- | --- |
| Urban-urban | -0.034 |
| Rural-rural | -0.013 |
| Urban-rural | -0.022 |

Table S12

Analysis of molecular variance (AMOVA) showing genetic variation sourced from three hierarchical levels when urbanization was classified based on Distance from the City Center and only sampling sites with at least two individuals were included. Also shown for each hierarchical level are degrees of freedom, sums of squares, mean squares, variances (sigma), percent variation, and p-values from permutation tests.

| Level | df | SS | MS | Sigma | Variation | p |
| --- | --- | --- | --- | --- | --- | --- |
| Among urban/rural habitats | 1 | 69.372 | 69.372 | -0.057 | -0.079% | 0.697 |
| Among sampling sites within urban/rural habitats | 43 | 3,164.149 | 73.585 | 0.749 | 1.05% | 0.019 |
| Within sampling sites | 134 | 9,462.892 | 70.619 | 70.619 | 99.03% | 0.017 |
| Total | 178 | 12,696.413 | 71.328 | 71.311 | 100% |  |

Table S13

Results of PERMANOVA testing whether genetic distances differed between urban and rural habitats. Shown are the metrics used to classify urbanization, predictor variables, degrees of freedom, sums of squares, partial R2, F-statistics, and p-values.

| Urbanization | Variable | df | SS | R2 | F | p |
| --- | --- | --- | --- | --- | --- | --- |
| Distance from the City Center | Distance from the City Center | 1 | 0.241 | 0.012 | 3.098 | 1.000 |
| Residual | 254 | 19.759 | 0.988 |  |  |
| Total | 255 | 20.000 | 1.000 |  |  |
| Urbanization Score | Urbanization Score | 1 | 0.238 | 0.012 | 3.055 | 1.000 |
| Residual | 254 | 19.762 | 0.988 |  |  |
| Total | 255 | 20.000 | 1.000 |  |  |

Table S14

Results of PERMDISP testing whether genetic distance variances differed between urban and rural habitats. Shown are the metrics used to classify urbanization, predictor variables, degrees of freedom, sums of squares, mean squares, F-statistics, and p-values. There were 150 urban and 106 rural individuals when urbanization was classified with Distance from the City Center, and 141 urban and 115 rural individuals when classified with Urbanization Score.

| Urbanization | Variable | df | SS | MS | F | p |
| --- | --- | --- | --- | --- | --- | --- |
| Distance from the City Center | Urban/Rural Habitats | 1 | 0.013 | 0.013 | 3.25 | 0.073 |
| Residuals | 254 | 1.019 | 0.004 |  |  |
| Urbanization Score | Urban/Rural Habitats | 1 | 0.007 | 0.007 | 1.812 | 0.180 |
| Residuals | 254 | 1.034 | 0.004 |  |  |

Table S15

Results from Mantel correlograms when urbanization was classified based on Distance from the City Center and the analysis was restricted to one individual per sampling site. Shown are urban and rural groups, spatial lags between distance classes, distance classes, the number of pairs per distance class, Mantel r from Pearson correlations, and p-values corrected with the Holm method that accounted for progressive multiple testing.

| Group | Spatial Lag (km) | Distance Class (km) | N | Mantel r | p |
| --- | --- | --- | --- | --- | --- |
| Urban | 2.5 | 1.25 | 64 | 0.000 | 0.461 |
|  |  | 3.75 | 158 | 0.002 | 0.909 |
|  |  | 6.25 | 308 | -0.021 | 0.647 |
|  |  | 8.75 | 288 | 0.008 | 1 |
|  |  | 11.25 | 340 | -0.018 | 1 |
|  |  | 13.75 | 294 | -0.024 | 0.995 |
|  |  | 16.25 | 330 | -0.045 | 0.266 |
|  |  | 18.75 | 298 | 0.025 | 1 |
|  |  | 21.25 | 296 | -0.039 | 0.315 |
|  |  | 23.75 | 242 | 0.010 | 1 |
|  |  | 26.25 | 248 | 0.032 | 0.89 |
|  |  | 28.75 | 160 | 0.032 | 0.919 |
|  |  | 31.25 | 174 | 0.032 | 0.967 |
|  |  | 33.75 | 118 | -0.033 | 1 |
| Urban | 10 | 5 | 818 | -0.008 | 0.43 |
| 15 | 1,262 | -0.037 | 0.256 |
| 25 | 946 | 0.015 | 0.615 |
| Rural | 10 | 5 | 204 | 0.029 | 0.192 |
| 15 | 482 | 0.035 | 0.252 |
| 25 | 436 | -0.007 | 0.45 |

Table S16

Results from memgene analysis when urbanization was classified based on Distance from the City Center and the analysis was restricted to one individual per sampling site for only rural sampling sites. Shown are the statistically significant memgene variables, the corresponding Moran’s Eigenvectors, and the amount of genetic variation explained by spatial patterns (R2). Adjusted R² for model: 0.01.

| Memgene Variable | Moran’s Eigenvector | R2 |
| --- | --- | --- |
| 1 | 4 | 0.71 |
| 2 | 2 | 0.29 |

Table S17

Results from Mantel correlograms when urbanization was classified based on Urbanization Score and the analysis was restricted to one individual per sampling site. Shown are urban and rural groups, spatial lags between distance classes, distance classes, the number of pairs per distance class, Mantel r from Pearson correlations, and p-values corrected with the Holm method that accounted for progressive multiple testing.

| Group | Spatial Lag (km) | Distance Class (km) | N | Mantel r | p |
| --- | --- | --- | --- | --- | --- |
| Urban | 2.5 | 1.25 | 48 | 0.025 | 0.154 |
|  |  | 3.75 | 130 | 0.038 | 0.138 |
|  |  | 6.25 | 274 | 0.035 | 0.248 |
|  |  | 8.75 | 250 | 0.060 | 0.06 |
|  |  | 11.25 | 284 | 0.023 | 0.372 |
|  |  | 13.75 | 264 | 0.027 | 0.496 |
|  |  | 16.25 | 282 | 0.002 | 0.619 |
|  |  | 18.75 | 250 | 0.051 | 0.154 |
|  |  | 21.25 | 232 | -0.007 | 0.821 |
|  |  | 23.75 | 192 | 0.008 | 1 |
|  |  | 26.25 | 206 | -0.002 | 1 |
|  |  | 28.75 | 148 | 0.038 | 0.71 |
|  |  | 31.25 | 146 | -0.016 | 1 |
|  |  | 33.75 | 140 | -0.032 | 1 |
| Urban | 10 | 5 | 702 | 0.088 | 0.019 |
| 15 | 1,080 | 0.060 | 0.048 |
| 25 | 778 | 0.018 | 0.257 |
| 35 | 468 | -0.071 | 0.076 |
| Rural | 10 | 5 | 210 | 0.011 | 0.349 |
| 15 | 532 | -0.013 | 0.619 |
| 25 | 478 | -0.009 | 0.929 |
| 35 | 486 | 0.019 | 1 |
| 45 | 408 | -0.039 | 0.624 |

Table S18

Results from memgene analysis when urbanization was classified based on Urbanization Score and the analysis was restricted to one individual per sampling site for only rural sampling sites. Shown are the statistically significant memgene variables, the corresponding Moran’s Eigenvectors, and the amount of genetic variation explained by spatial patterns (R2). Adjusted R² for model: 0.0228

| Memgene Variable | Moran’s Eigenvector | R2 |
| --- | --- | --- |
| 1 | 3 | 0.511 |
| 2 | 4 | 0.191 |
| 3 | 14 | 0.174 |
| 4 | 8 | 0.124 |

Table S19

Results from Mantel correlograms when urbanization was classified based on Distance from the City Center and all genotyped individuals were included. Shown are urban and rural groups, spatial lags between distance classes, distance classes, the number of pairs per distance class, Mantel r from Pearson correlations, and p-values corrected with the Holm method that accounted for progressive multiple testing.

| Group | Spatial Lag (km) | Distance Class (km) | N | Mantel r | p |
| --- | --- | --- | --- | --- | --- |
| Urban | 2.5 | 1.25 | 1,206 | 0.003 | 0.418 |
|  |  | 3.75 | 1,514 | -0.017 | 0.336 |
|  |  | 6.25 | 2,904 | -0.005 | 0.761 |
|  |  | 8.75 | 2,130 | -0.010 | 0.86 |
|  |  | 11.25 | 2,266 | -0.018 | 0.764 |
|  |  | 13.75 | 1,528 | -0.006 | 1 |
|  |  | 16.25 | 2,146 | -0.011 | 1 |
|  |  | 18.75 | 1,536 | -0.008 | 1 |
|  |  | 21.25 | 1,754 | 0.000 | 1 |
|  |  | 23.75 | 1,176 | 0.006 | 1 |
|  |  | 26.25 | 984 | 0.003 | 1 |
|  |  | 28.75 | 524 | 0.003 | 1 |
|  |  | 31.25 | 564 | 0.009 | 1 |
|  |  | 33.75 | 452 | 0.010 | 1 |
| Urban | 10 | 5 | 7,754 | -0.017 | 0.243 |
| 15 | 7,476 | -0.026 | 0.2 |
| 25 | 4,438 | 0.007 | 0.486 |
| Rural | 10 | 5 | 1,740 | 0.056 | 0.047 |
| 15 | 2,210 | 0.013 | 0.361 |
| 25 | 1,476 | 0.032 | 0.208 |

Table S20

Results from Mantel correlograms when urbanization was classified based on Urbanization Score and all genotyped individuals were included. Shown are urban and rural groups, spatial lags between distance classes, distance classes, the number of pairs per distance class, Mantel r from Pearson correlations, and p-values corrected with the Holm method that accounted for progressive multiple testing.

| Group | Spatial Lag (km) | Distance Class (km) | N | Mantel r | p |
| --- | --- | --- | --- | --- | --- |
| Urban | 2.5 | 1.25 | 900 | 0.008 | 0.257 |
|  |  | 3.75 | 1,086 | -0.001 | 0.513 |
|  |  | 6.25 | 2,682 | 0.012 | 0.77 |
|  |  | 8.75 | 1,970 | 0.008 | 1 |
|  |  | 11.25 | 1,956 | -0.004 | 1 |
|  |  | 13.75 | 1,434 | -0.005 | 1 |
|  |  | 16.25 | 1,840 | -0.008 | 1 |
|  |  | 18.75 | 1,302 | 0.002 | 1 |
|  |  | 21.25 | 1,320 | 0.032 | 0.279 |
|  |  | 23.75 | 1,004 | 0.003 | 1 |
|  |  | 26.25 | 952 | 0.014 | 1 |
|  |  | 28.75 | 486 | -0.014 | 1 |
|  |  | 31.25 | 490 | -0.013 | 1 |
|  |  | 33.75 | 430 | -0.001 | 1 |
| Urban | 10 | 5 | 6,638 | 0.017 | 0.247 |
| 15 | 6,532 | -0.009 | 0.494 |
| 25 | 3,762 | 0.025 | 0.45 |
| 35 | 1,476 | -0.019 | 0.731 |
| Rural | 10 | 5 | 1,948 | -0.016 | 0.289 |
| 15 | 2,618 | 0.037 | 0.188 |
| 25 | 1,778 | 0.030 | 0.234 |
| 35 | 1,516 | -0.049 | 0.128 |
| 45 | 1,212 | -0.047 | 0.232 |

Table S21

Results from Mantel correlograms for the entire population and the analysis was restricted to one individual per sampling site. Shown are spatial lags between distance classes, distance classes, the number of pairs per distance class, Mantel r from Pearson correlations, and p-values corrected with the Holm method that accounted for progressive multiple testing. 

| Spatial Lag (km) | Distance Class (km) | N | Mantel r | p |
| --- | --- | --- | --- | --- |
| 2.5 | 1.25 | 74 | 0.006 | 0.333 |
|  | 3.75 | 202 | 0.002 | 0.665 |
|  | 6.25 | 406 | -0.001 | 0.998 |
|  | 8.75 | 418 | -0.001 | 1 |
|  | 11.25 | 524 | -0.005 | 1 |
|  | 13.75 | 484 | 0.007 | 1 |
|  | 16.25 | 524 | -0.011 | 1 |
|  | 18.75 | 580 | 0.015 | 1 |
|  | 21.25 | 560 | -0.007 | 1 |
|  | 23.75 | 542 | 0.005 | 1 |
|  | 26.25 | 590 | -0.004 | 1 |
|  | 28.75 | 572 | -0.006 | 1 |
|  | 31.25 | 600 | 0.007 | 1 |
|  | 33.75 | 618 | -0.020 | 1 |
|  | 38.75 | 596 | 0.002 | 1 |
| 10 | 5 | 1,100 | 0.002 | 0.475 |
| 15 | 2,112 | 0.003 | 0.845 |
| 25 | 2,264 | -0.007 | 1 |
| 35 | 2,406 | -0.014 | 0.811 |
| 45 | 2,228 | -0.019 | 0.534 |
| 55 | 1,654 | -0.008 | 1 |
| 65 | 1,128 | 0.001 | 1 |

Table S22

Results from Mantel correlograms for the entire population when all genotyped individuals were included. Shown are spatial lags between distance classes, distance classes, the number of pairs per distance class, Mantel r from Pearson correlations, and p-values corrected with the Holm method that accounted for progressive multiple testing.

| Spatial Lag (km) | Distance Class (km) | N | Mantel r | p |
| --- | --- | --- | --- | --- |
| 2.5 | 1.25 | 1,566 | 0.093 | 0.001 |
|  | 3.75 | 1,874 | -0.003 | 0.404 |
|  | 6.25 | 3,764 | -0.009 | 0.505 |
|  | 8.75 | 3,454 | -0.006 | 0.758 |
|  | 11.25 | 3,302 | 0.001 | 1 |
|  | 13.75 | 2,728 | -0.001 | 1 |
|  | 16.25 | 3,476 | -0.003 | 1 |
|  | 18.75 | 3,074 | -0.008 | 1 |
|  | 21.25 | 3,026 | -0.010 | 1 |
|  | 23.75 | 2,652 | -0.002 | 1 |
|  | 26.25 | 2,834 | -0.008 | 1 |
|  | 28.75 | 2,488 | -0.011 | 1 |
|  | 31.25 | 2,412 | 0.003 | 1 |
|  | 33.75 | 2,722 | -0.019 | 0.377 |
|  | 38.75 | 2,260 | -0.011 | 1 |
| 10 | 5 | 10,658 | 0.027 | 0.078 |
| 15 | 12,580 | -0.006 | 0.368 |
| 25 | 11,000 | -0.017 | 0.234 |
| 35 | 9,684 | -0.018 | 0.296 |
| 45 | 8,452 | -0.016 | 0.37 |
| 55 | 5,486 | -0.001 | 0.735 |
| 65 | 3,278 | 0.024 | 0.245 |

Table S23

Results from memgene analysis for the entire population when all genotyped individuals were included. Shown are the statistically significant memgene variables, the corresponding Moran’s Eigenvectors, and the amount of genetic variation explained by spatial patterns (R2). Adjusted R² for model: 0.028.

| Memgene Variable | Moran’s Eigenvector | R2 |
| --- | --- | --- |
| 1 | 12 | 0.192 |
| 2 | 20 | 0.067 |
| 3 | 5 | 0.063 |
| 4 | 17 | 0.060 |
| 5 | 3 | 0.059 |
| 6 | 39 | 0.057 |
| 7 | 18 | 0.054 |
| 8 | 2 | 0.051 |
| 9 | 40 | 0.044 |
| 10 | 14 | 0.043 |
| 11 | 19 | 0.042 |
| 12 | 6 | 0.040 |
| 13 | 21 | 0.037 |
| 14 | 42 | 0.035 |
| 15 | 16 | 0.033 |
| 16 | 4 | 0.030 |
| 17 | 29 | 0.029 |
| 18 | 45 | 0.025 |
| 19 | 34 | 0.023 |
| 20 | 28 | 0.017 |
|  |

Table S19

Results of the breakpoint analysis identifying periods with distinct rates of change in the reconstructed trends in Ne over time. Each row represents a period. Shown are the years between the last year of sampling (2019) and the period’s starting year (i.e., years ago; ya), the period start, the median Ne at the start, the period end, the estimated Ne rate of change, the 95% confidence intervals, and the historical interpretation of concurrent events in the GTA.

| Years ago | Start | Start Ne | End | Rate | 95% CI (Lower) | 95% CI  (Upper) | Historical Interpretation |
| --- | --- | --- | --- | --- | --- | --- | --- |
| 2 | 2017 | 310,108.1 | 2018 | -1.776 | -1.676 | -1.879 | Conservation groups (e.g., Suzuki Foundation) encourage Toronto residents to plant milkweed |
| 9 | 2010 | 1,668,790.8 | 2017 | -0.267 | -0.259 | -0.275 | " |
| 42 | 1977 | 7,574,755.9 | 2010 | -0.049 | -0.048 | -0.051 | Urban expansion in Toronto |
| 194 | 1825 | 24,813,333.2 | 1977 | -0.007 | -0.007 | -0.008 | Colonization and settlement by Europeans; urban and rural expansion |
| 376 | 1643 | 46,700,202.9 | 1825 | -0.003 | -0.003 | -0.004 | European diseases decimate Indigenous populations |
| 792 | 1227 | 61,215,387.5 | 1643 | -6.80×10-4 | -5.50×10-4 | -8.10×10-4 | Indigenous agriculture grows |
| 2094 | -75 | 70,485,753.0 | 1227 | -1.10×10-4 | -8.00×10-5 | -1.30×10-4 | Indigenous populations present |
| 7742 | -5723 | 63,853,805.9 | -75 | 1.00×10-5 | 2.00×10-5 | 1.00×10-5 | " |
| 15000 | -12981 | 25,722,039.5 | -5723 | 1.40×10-4 | 1.40×10-4 | 1.30×10-4 | Glaciers retreat; climate warms; Indigenous populations present |

References

[Anderson, M. J. 2001. A new method for non‐parametric multivariate analysis of variance. Austral Ecol. 26:32–46.](http://paperpile.com/b/z5FcPm/KYG64)

[Hanlon, V. C. T., S. P. Otto, and S. N. Aitken. 2019. Somatic mutations substantially increase the per-generation mutation rate in the conifer ](http://paperpile.com/b/z5FcPm/6GlX)[Picea sitchensis](http://paperpile.com/b/z5FcPm/6GlX)[. Evol. Lett. 3:348–358.](http://paperpile.com/b/z5FcPm/6GlX)

Holm, S. 1979. A Simple Sequentially Rejective Multiple Test Procedure. Scand. J. Statist. 6:65–70.

[Jombart, T. 2008. Adegenet: A R package for the multivariate analysis of genetic markers. Bioinformatics 24:1403–1405.](http://paperpile.com/b/z5FcPm/tquYf)

[Jombart, T., S. Devillard, and F. Balloux. 2010. Discriminant analysis of principal components: a new method for the analysis of genetically structured populations. BMC Genet. 11:94.](http://paperpile.com/b/z5FcPm/x6SZy)

[Oksanen, J., G. L. Simpson, F. G. Blanchet, R. Kindt, P. Legendre, P. R. Minchin et al. 2019. vegan: community ecology package. R package version 2.5-2.](http://paperpile.com/b/z5FcPm/5eZC)
